# Supplementary material for: Biotransformation of Modified Benzylisoquinoline Alkaloids: Boldine and Berberine and In Silico Molecular Docking Studies of Metabolites on Telomerase and Human Protein Tyrosine Phosphatase 1B
Source: Pharmaceuticals (Basel). 2022 Sep 28;15(10):1195. doi: 10.3390/ph15101195 (PMC9611891; doi:10.3390/ph15101195)
Supplement: Supplementary file 1 [file pharmaceuticals-15-01195-s001.zip › pharmaceuticals-1930456-supplementary.pdf]

Supporting information

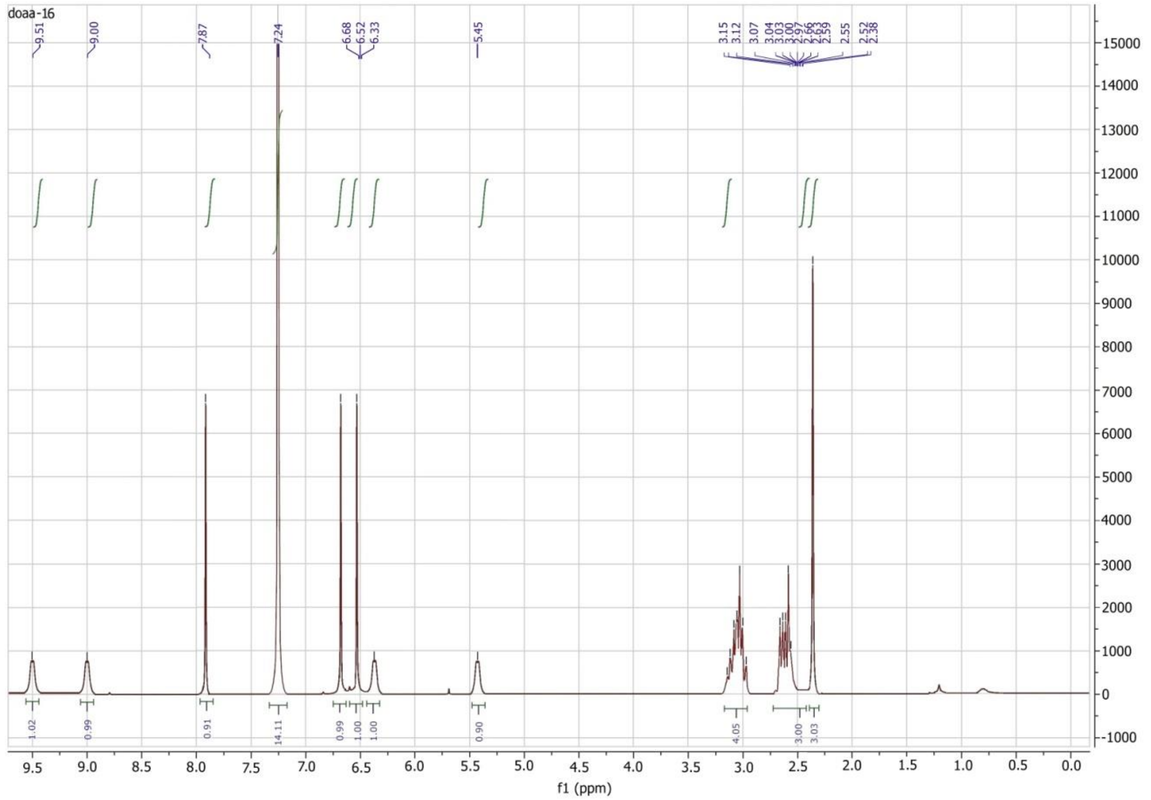

S1: ^{1}H NMR spectrum of boldine metabolite-**1** (400 MHz, $CDCl_3$).

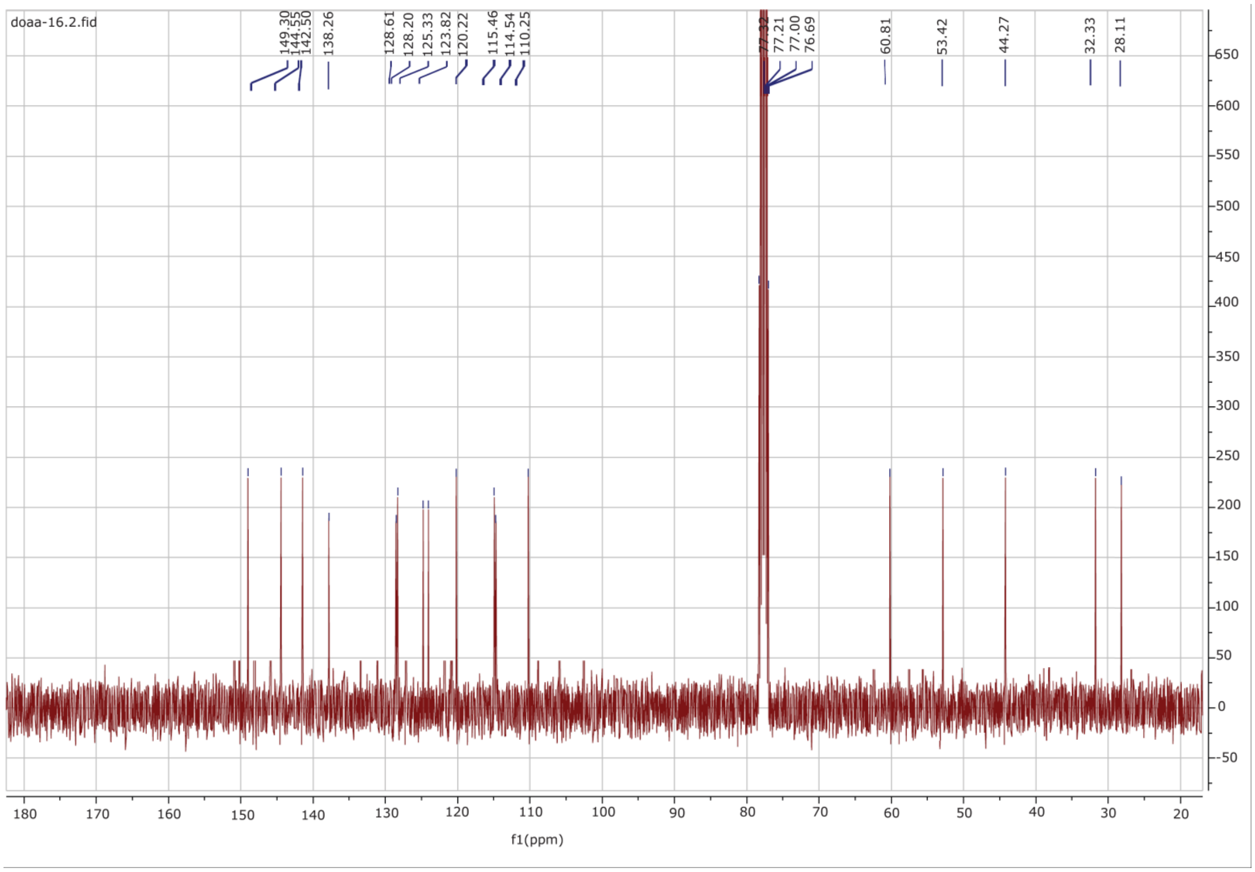

S2: ^{13}C NMR spectrum of boldine metabolite-**1** (100 MHz, $CDCl_3$).

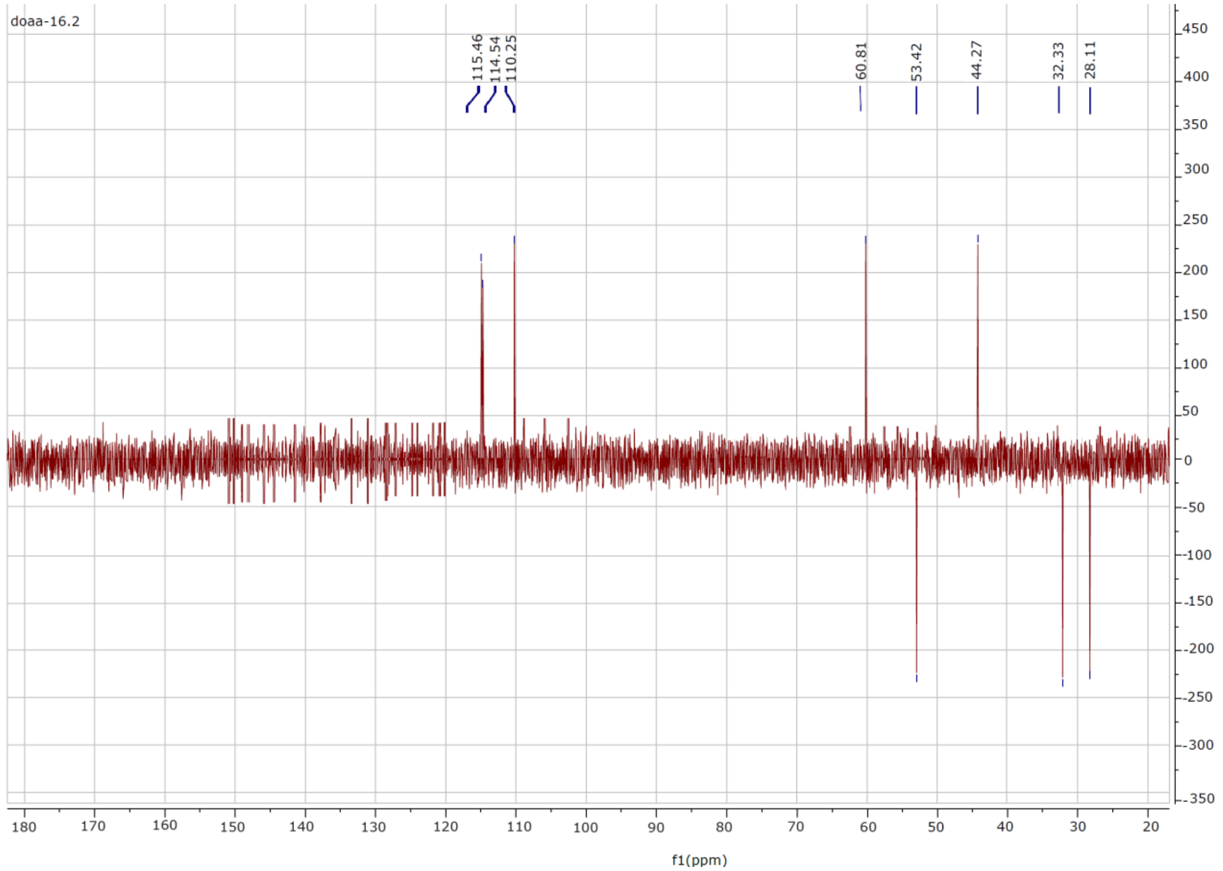

S3: DEPT 135 spectrum of boldine metabolite-**1** (100 MHz, $CDCl_3$).

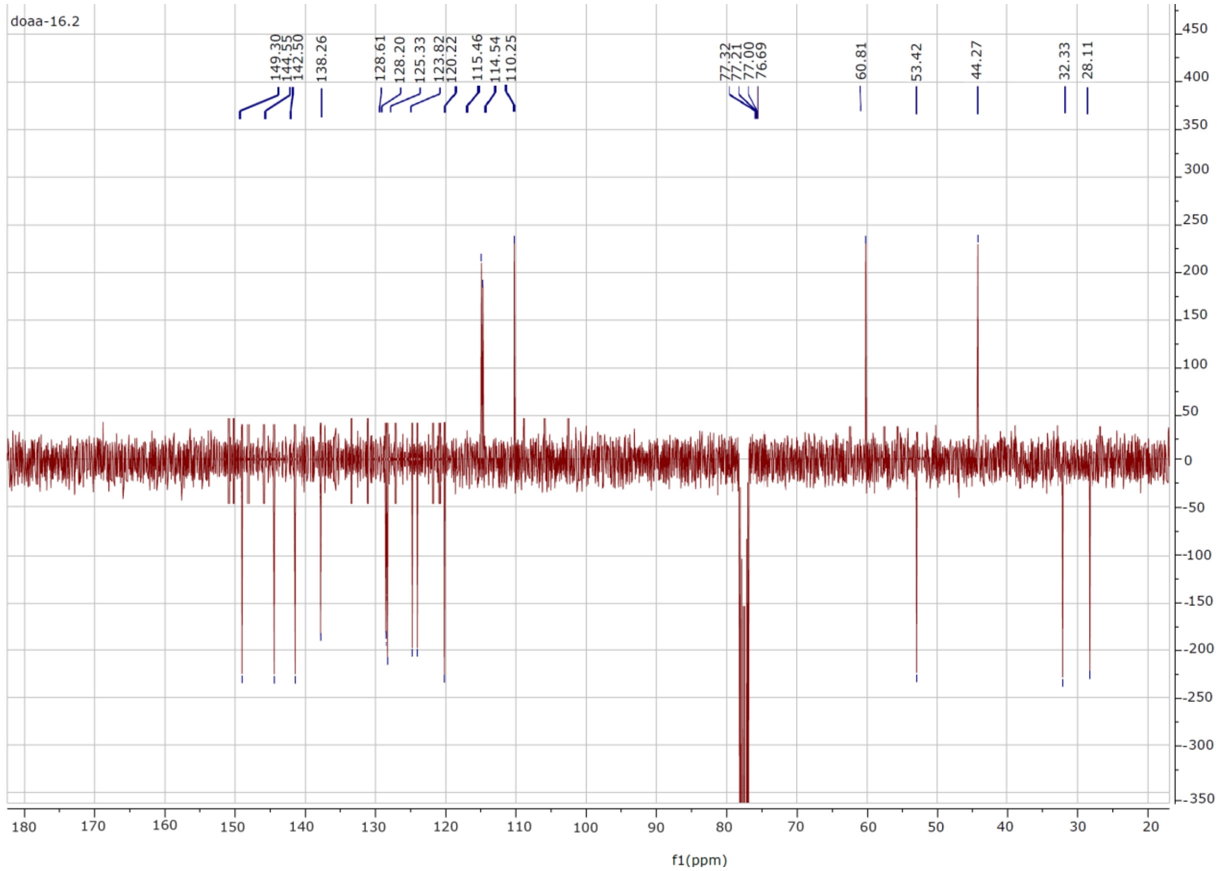

S4: APT spectrum of boldine metabolite-1 (100 MHz, CDCl3).

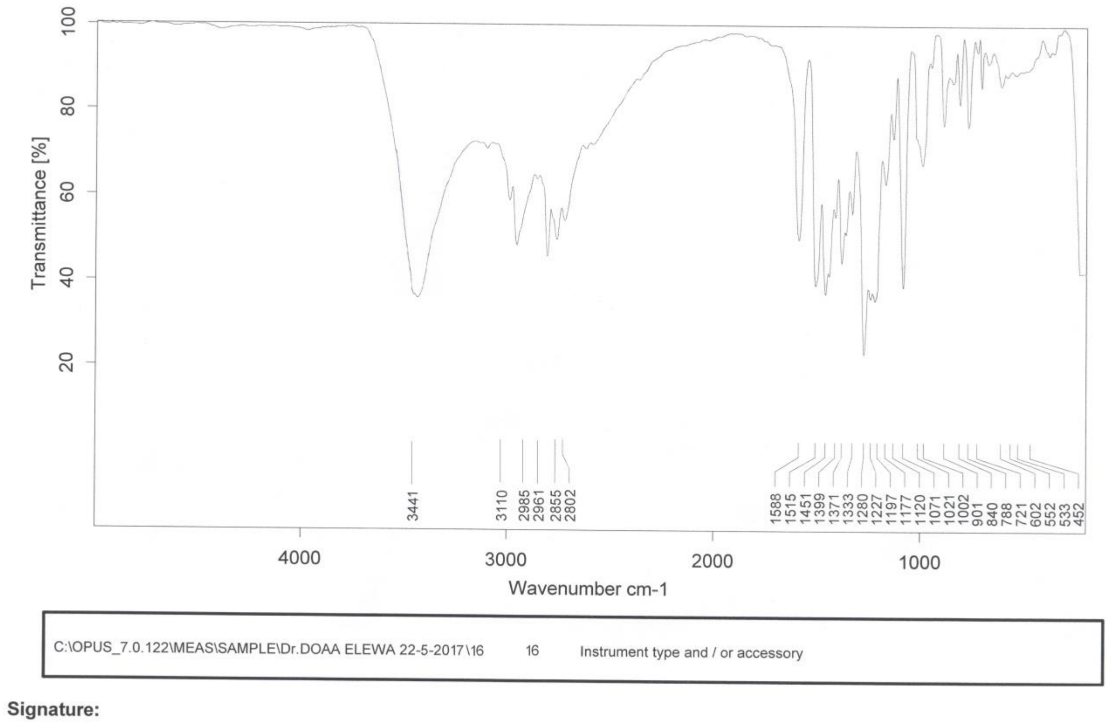

S5: IR spectrum of boldine metabolite-**1.**

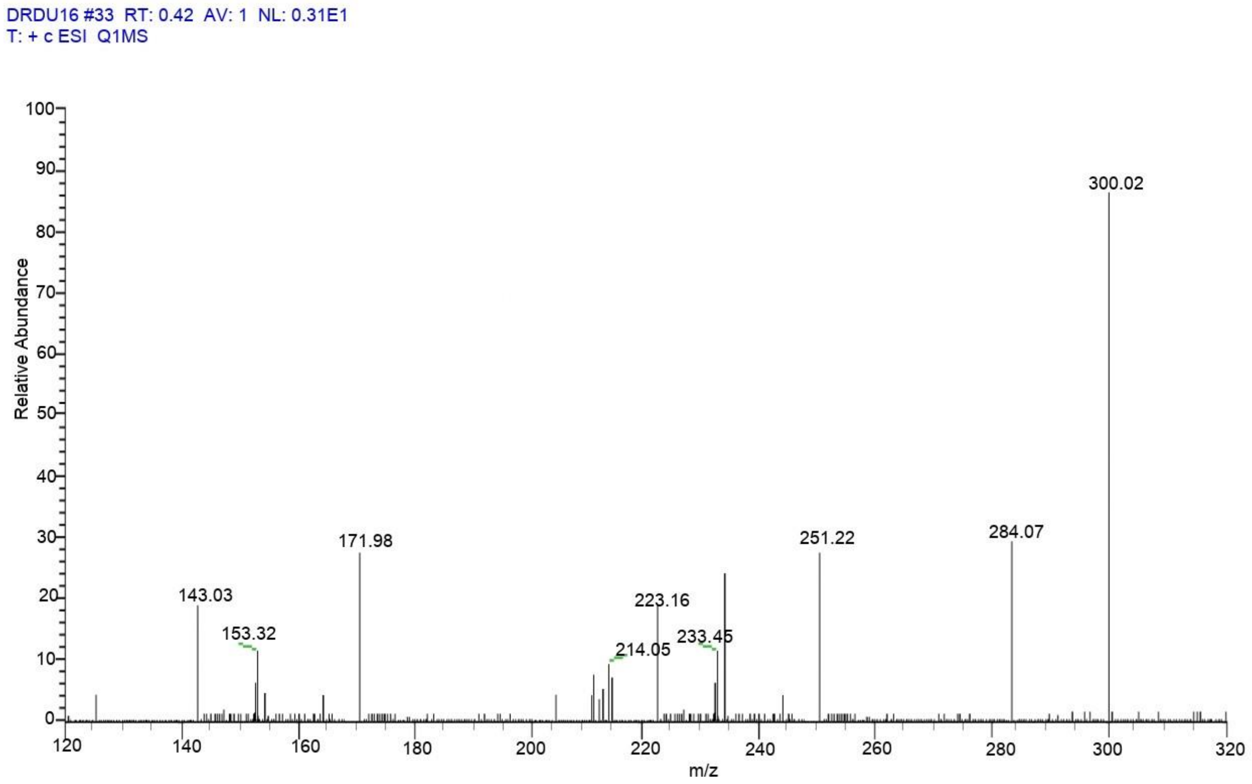

S6: (+) ESI-MS spectrum of boldine metabolite **1**.

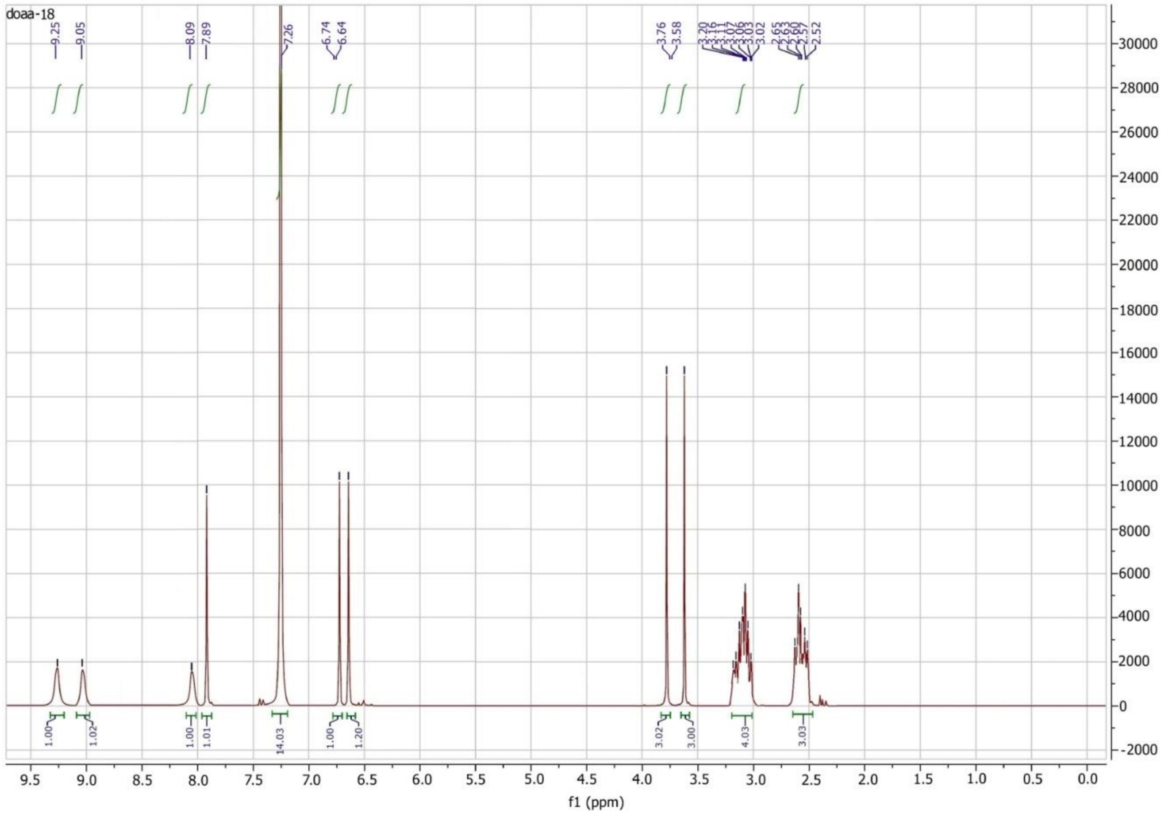

S7: ^{1}H NMR spectrum of boldine metabolite-**2** (400 MHz, $CDCl_3$).

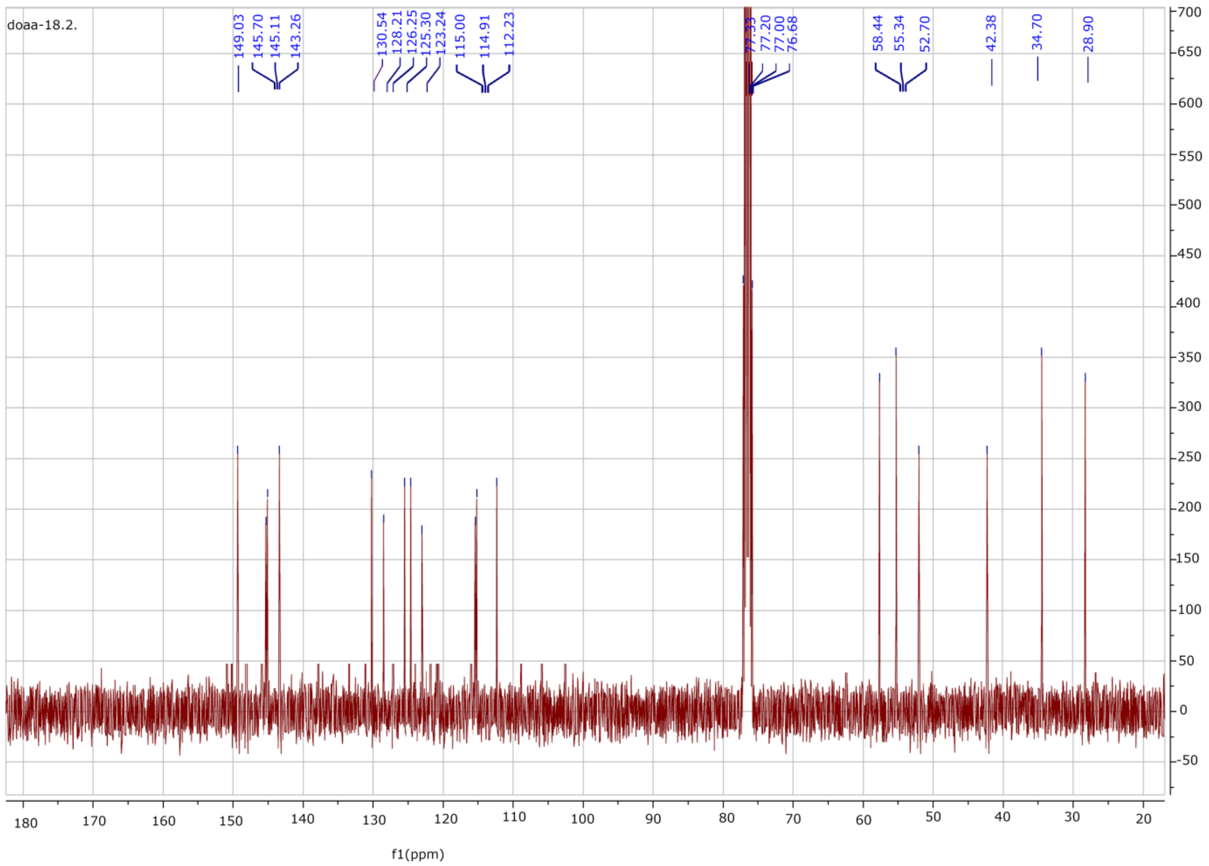

S8: ^{13}C NMR spectrum of boldine metabolite-**2** (100 MHz, $CDCl_3$).

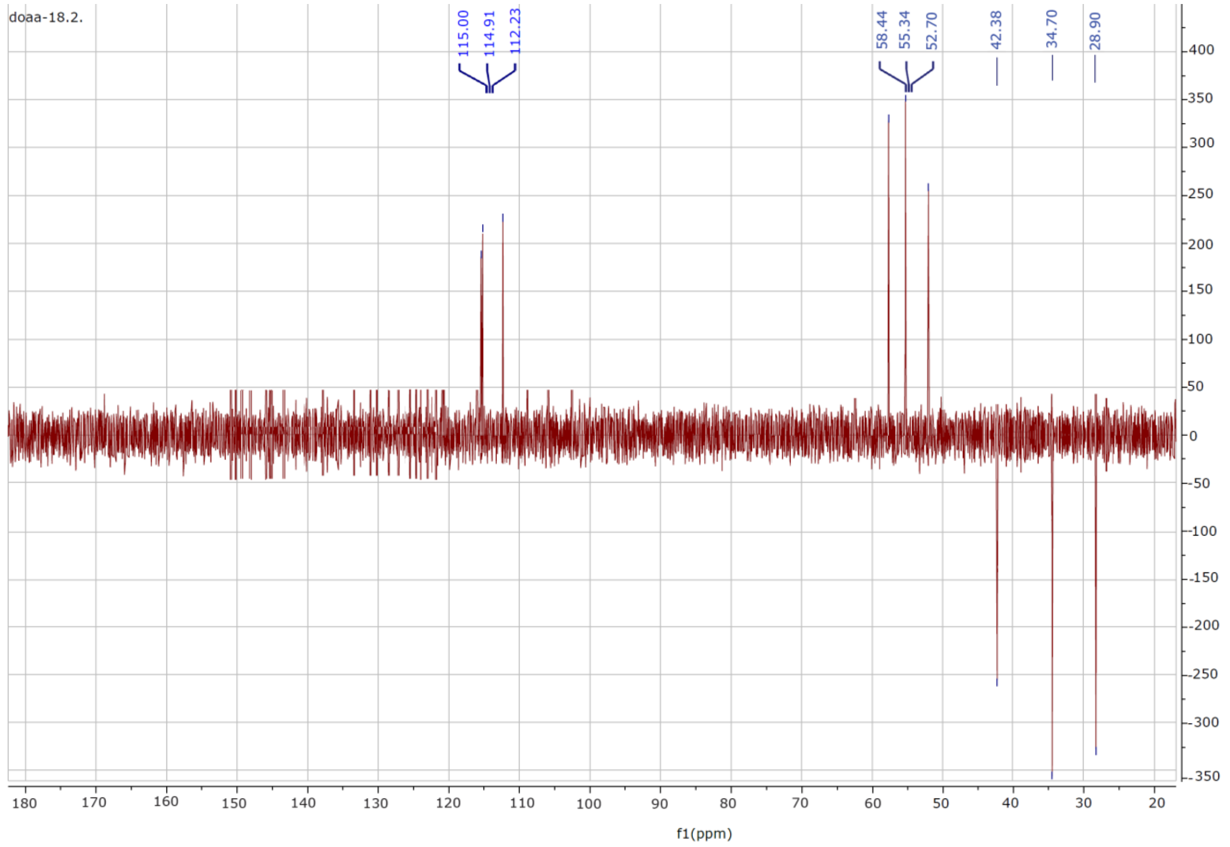

S9: DEPT 135 spectrum of boldine metabolite-**2** (100 MHz, $CDCl_3$).

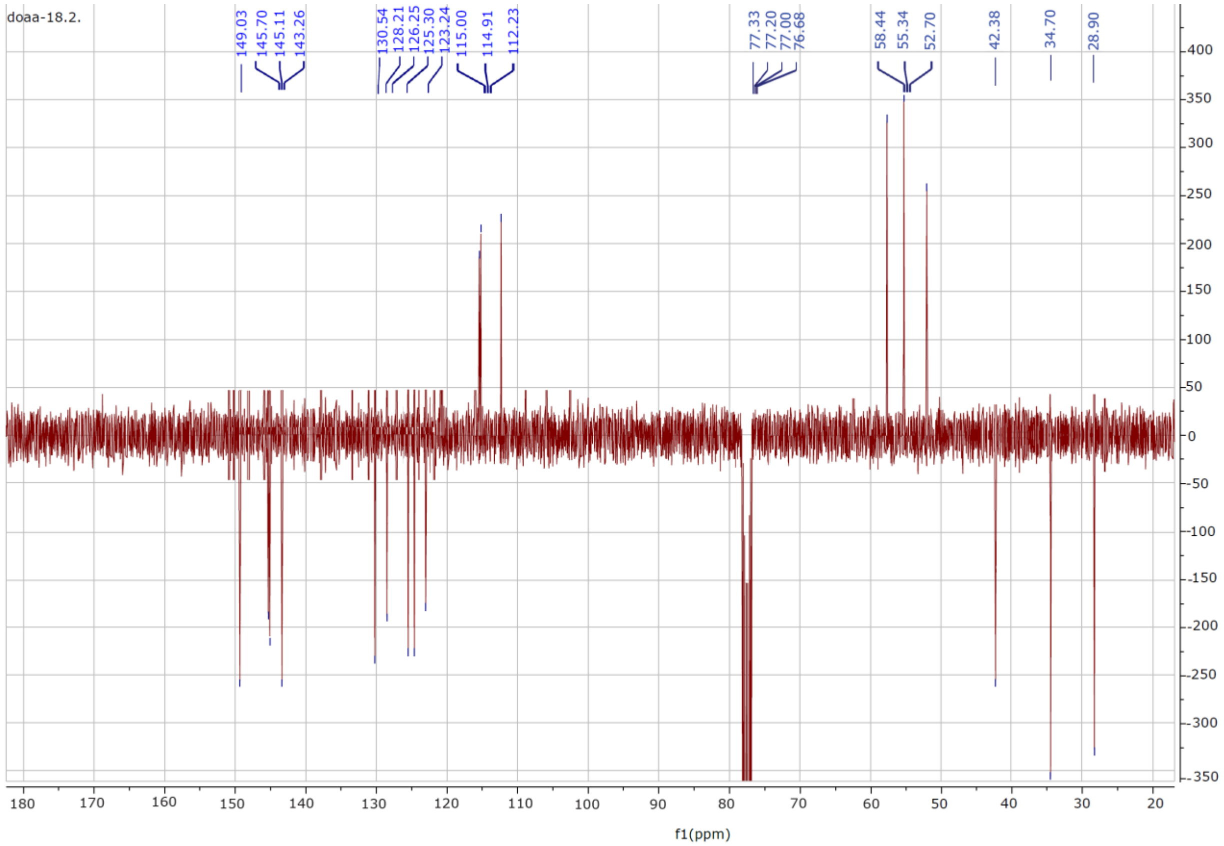

S10: APT spectrum of boldine metabolite-**2** ($CDCl_3$).

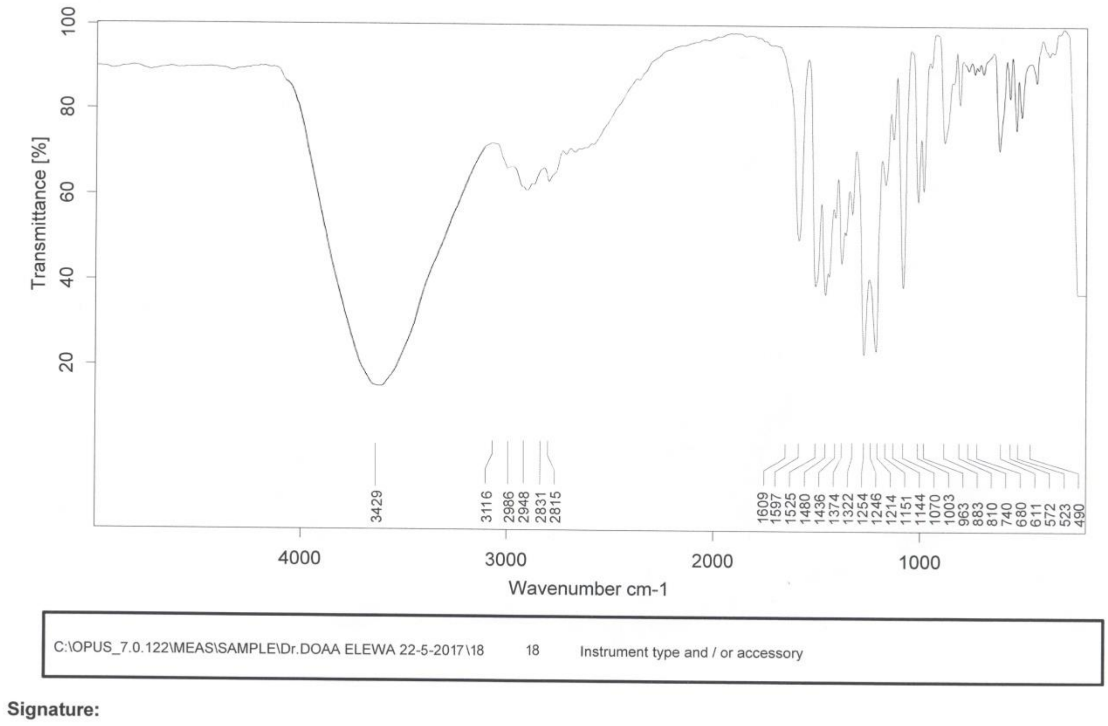

S11: IR spectrum of boldine metabolite **-2.**

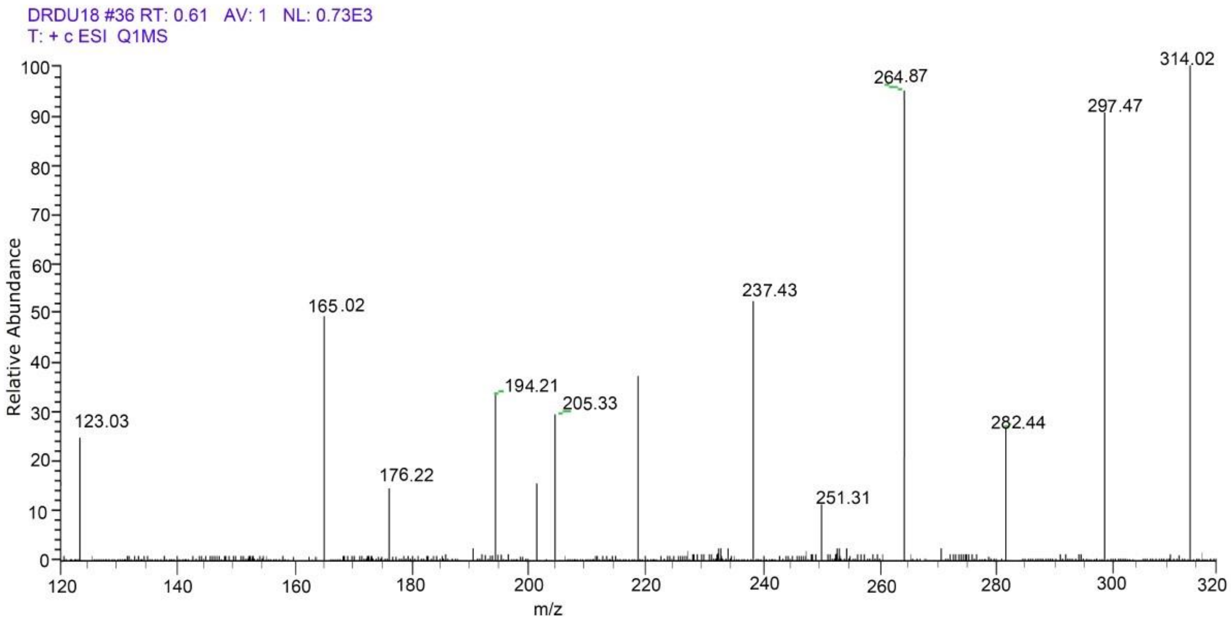

S12: (+) ESI- MS spectrum of boldine metabolite -**2**.

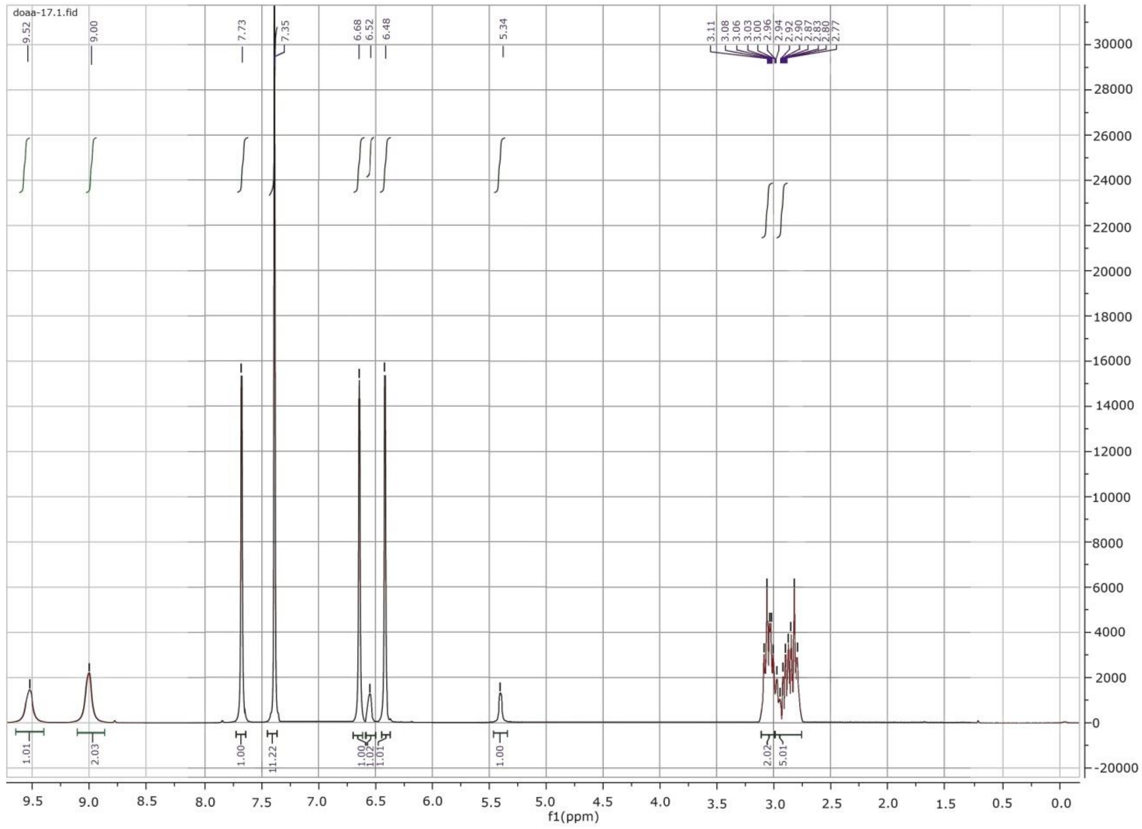

S13: ^{1}H NMR spectrum of boldine metabolite-**3** (400 MHz, $CDCl_3$).

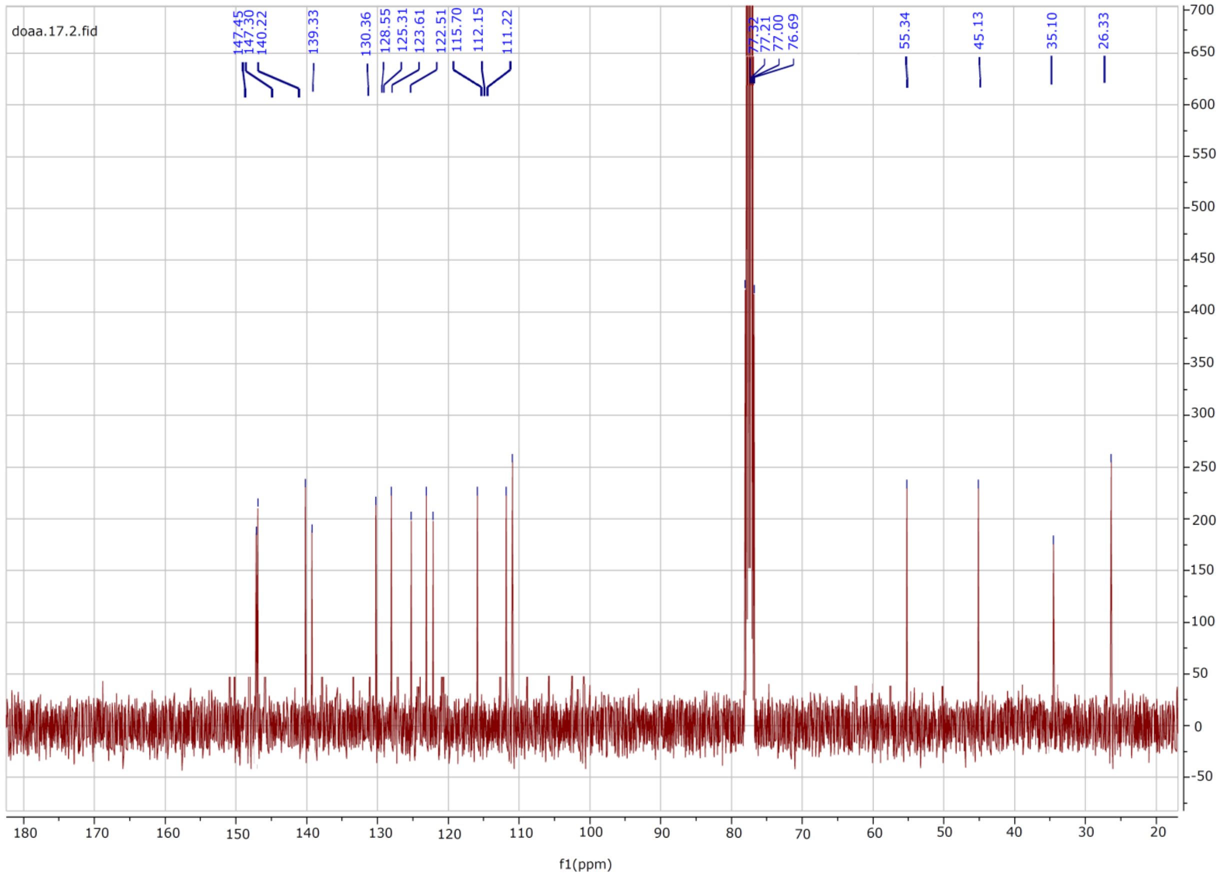

S14: ^{13}C NMR spectrum of boldine metabolite-**3** (100 MHz, $CDCl_3$).

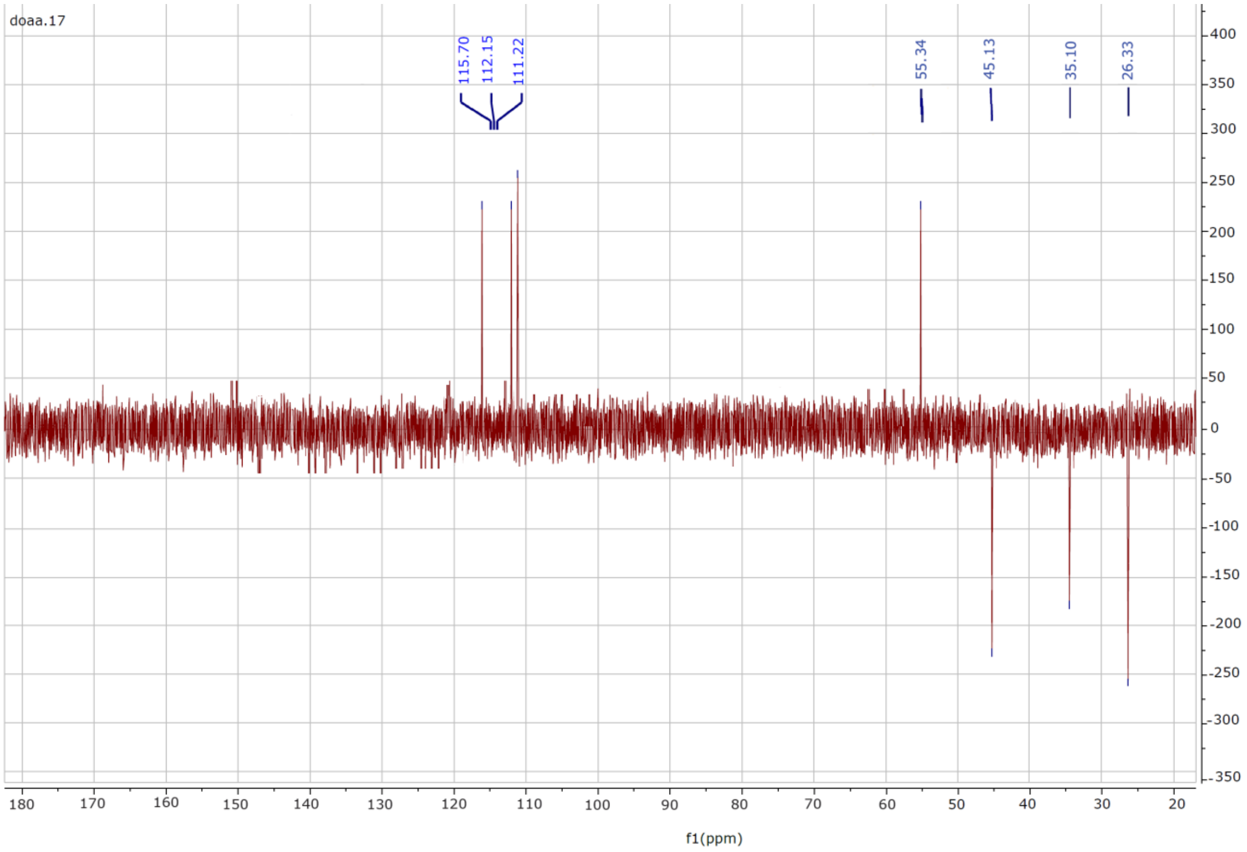

S15: DEPT 135 spectrum of boldine metabolite-**3** (100 MHz, $CDCl_3$).

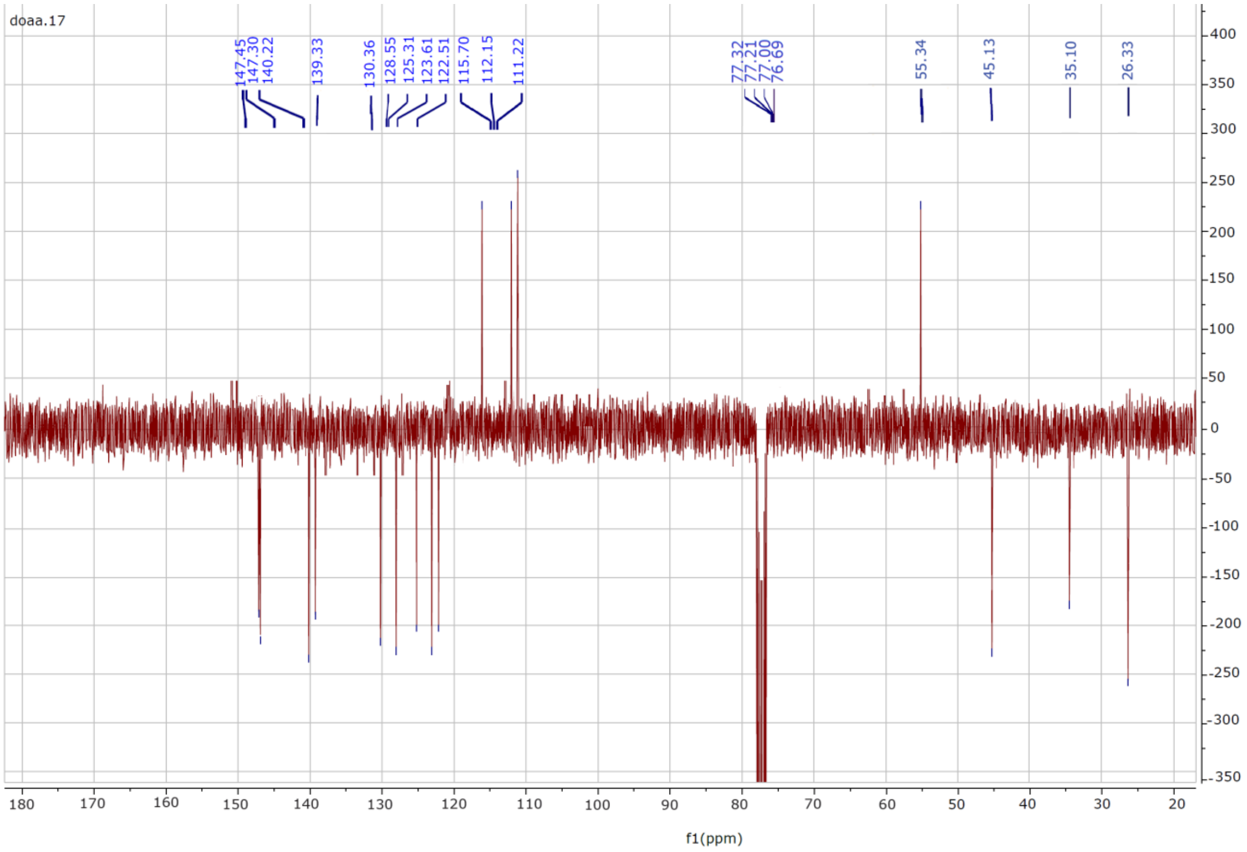

S16: APT spectrum of boldine metabolite-**3** ($CDCl_3$).

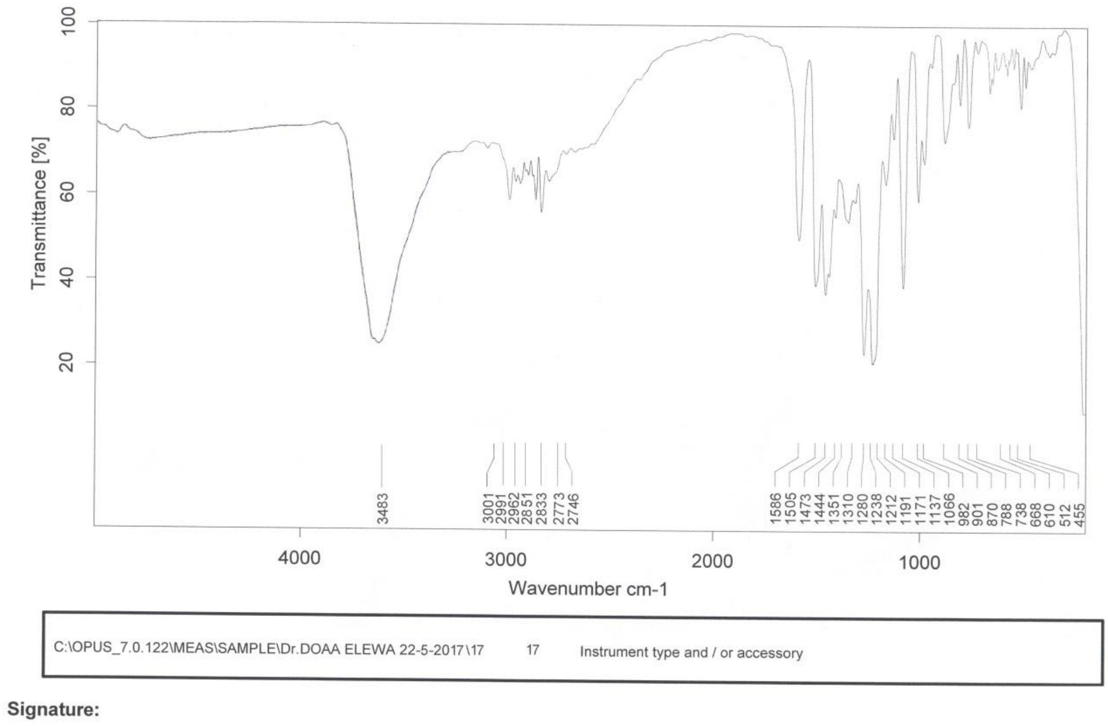

S17: IR spectrum of boldine metabolite **-3.**

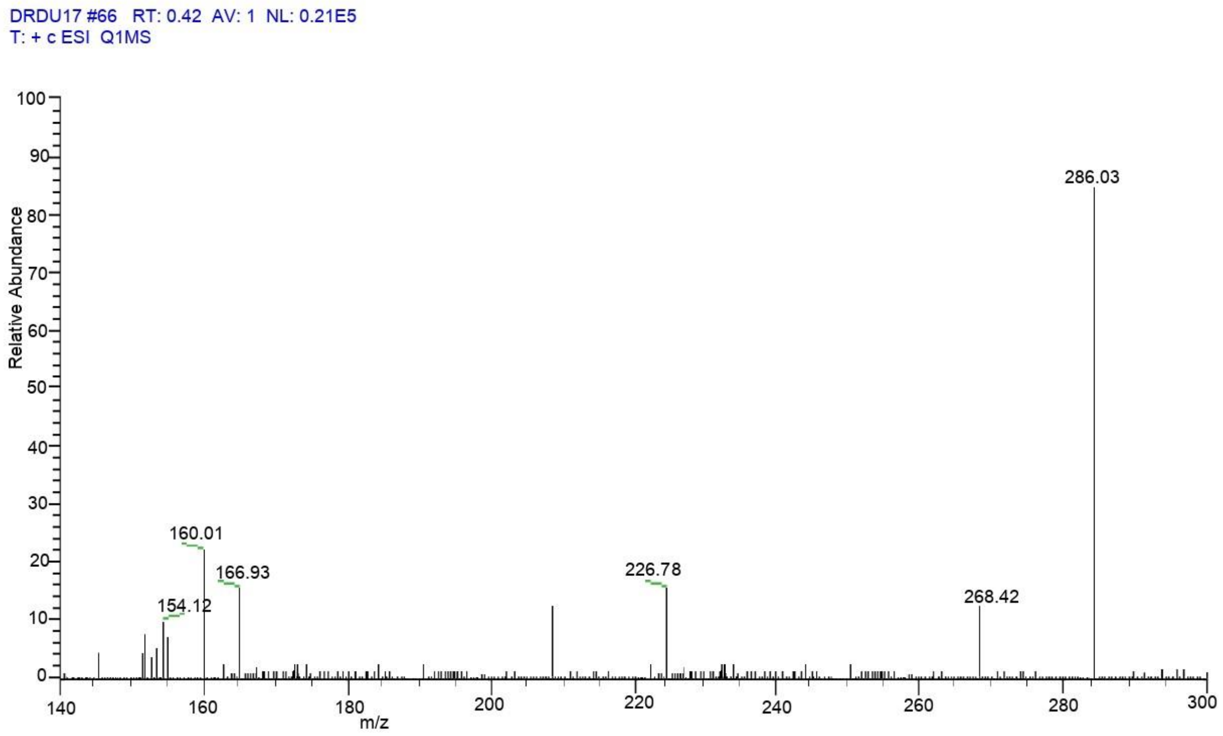

S18: (+) ESI- MS spectrum of boldine metabolite -**3**.

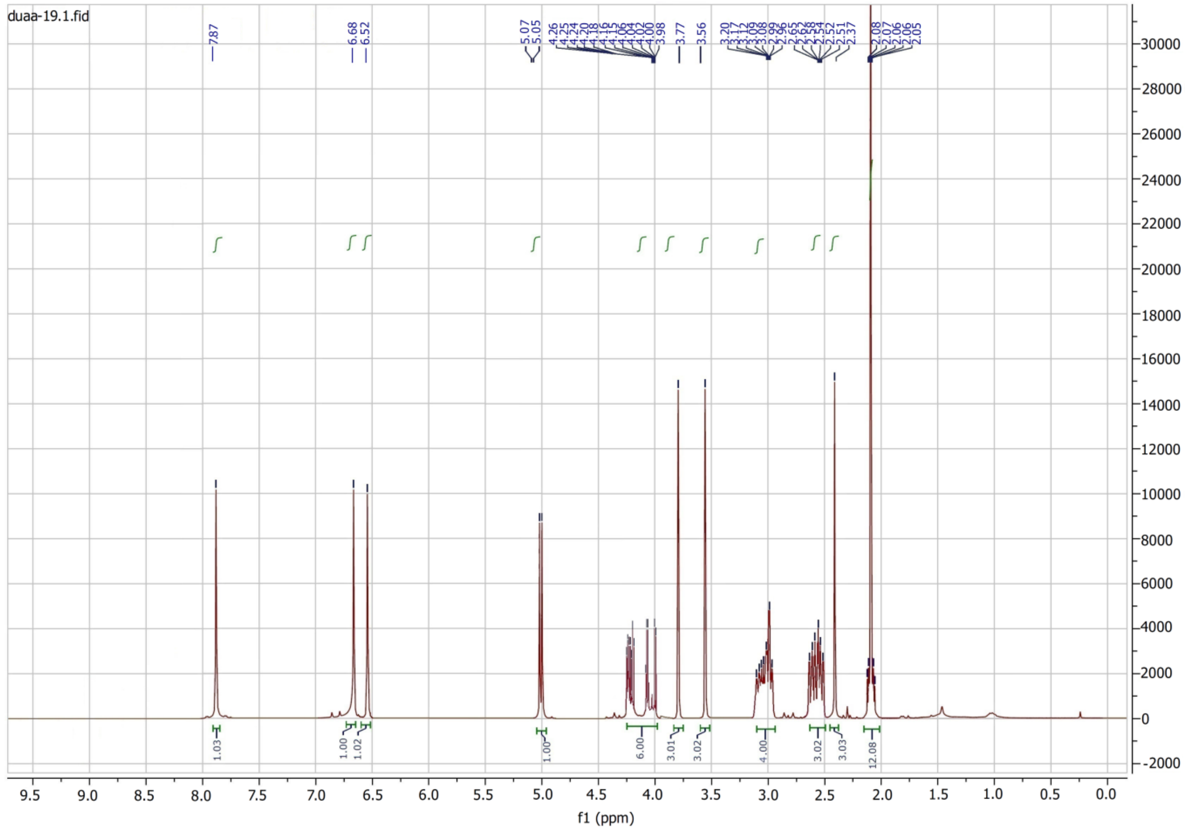

S19: ^{1}H NMR spectrum of boldine metabolite-**4** (400 MHz, Acetone-*d6*).

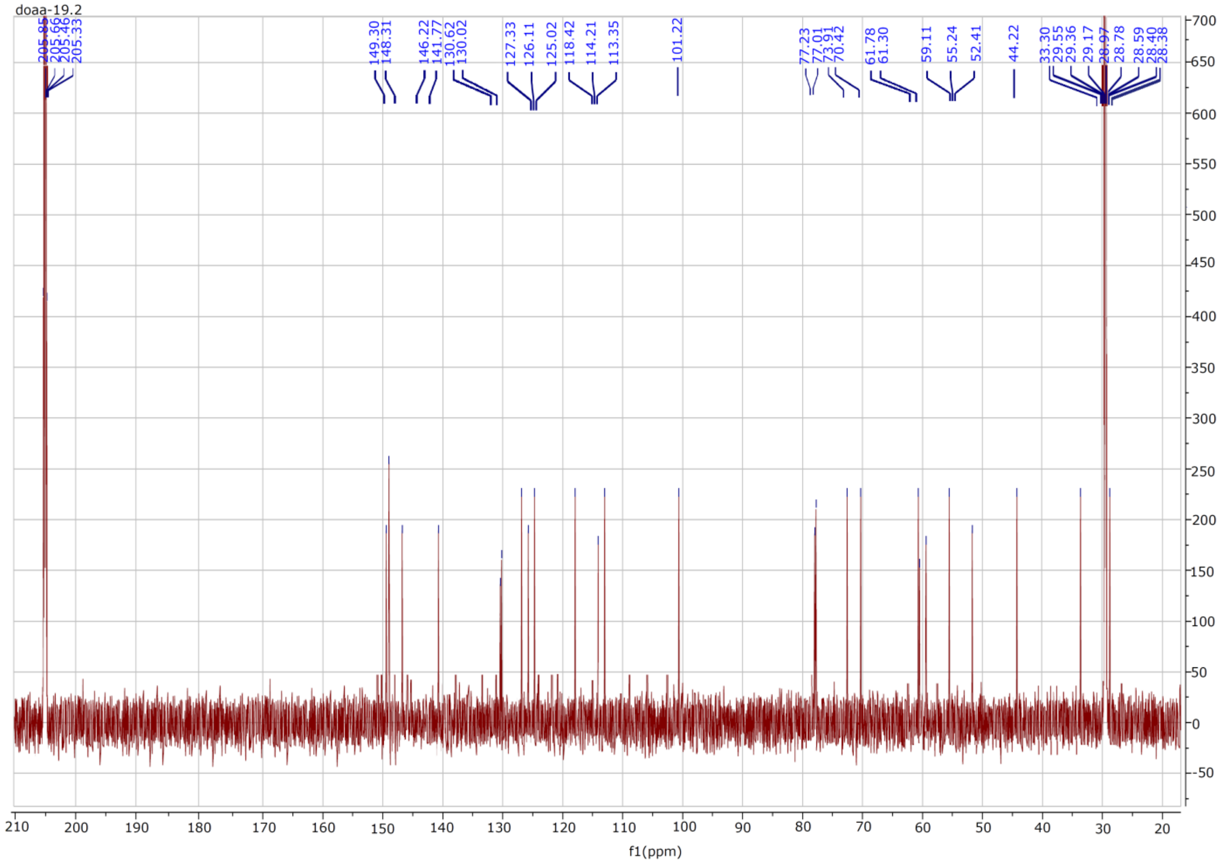

S20: ^{13}C NMR spectrum of boldine metabolite-**4** (100 MHz, Acetone-*d6*).

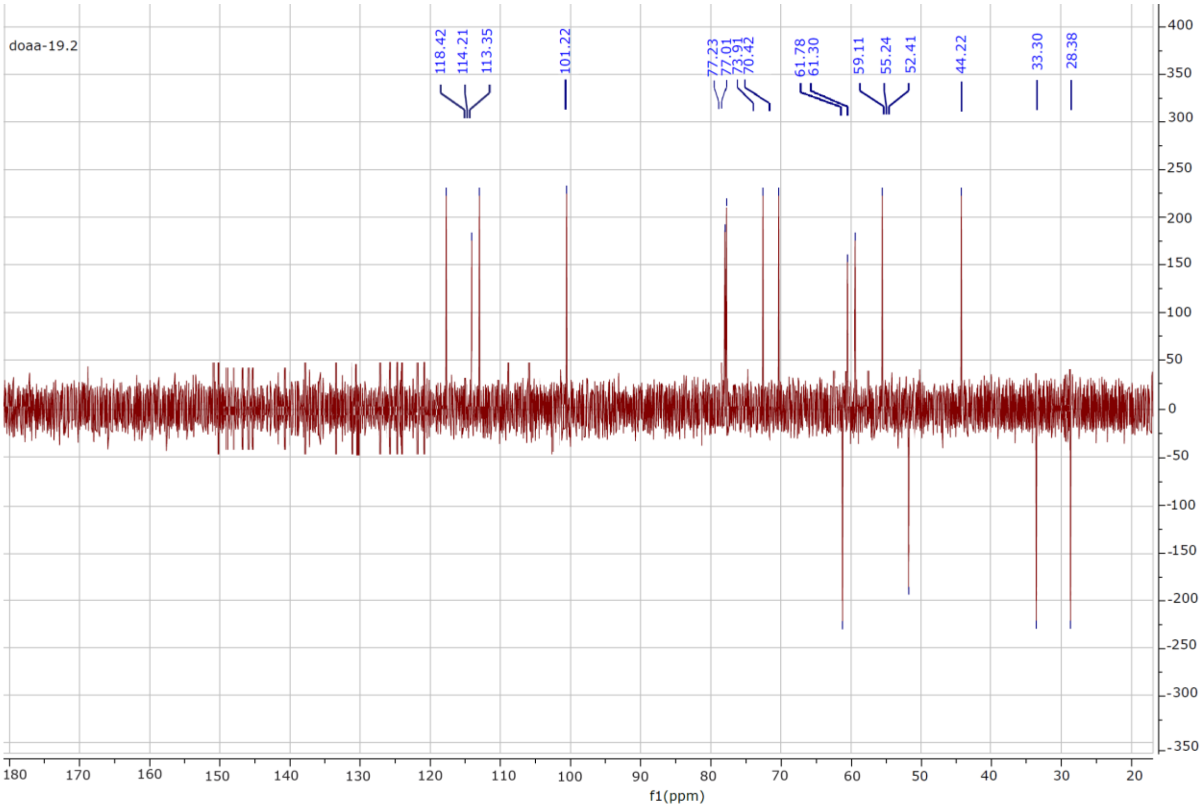

S21: DEPT135 spectrum of boldine metabolite-**4** (100 MHz, Acetone-*d6*).

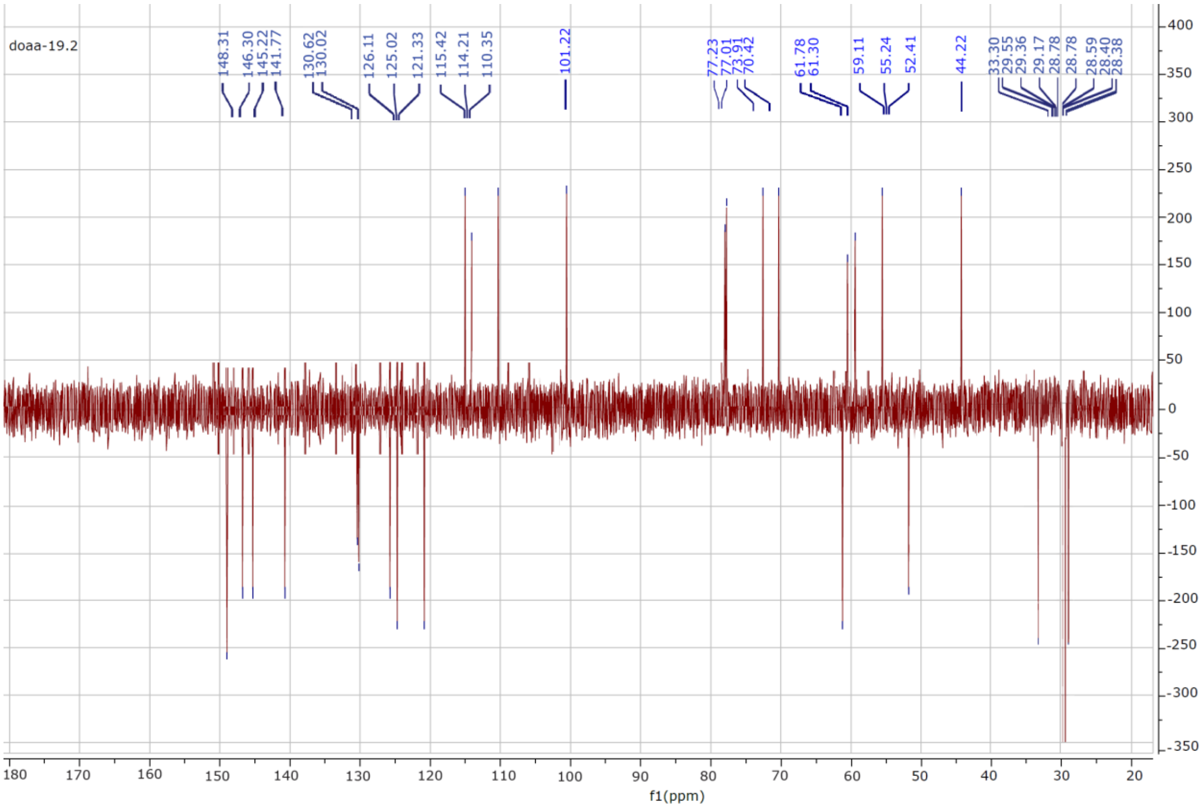

S22: APT spectrum of boldine metabolite-**4** (100 MHz, Acetone-*d6*).

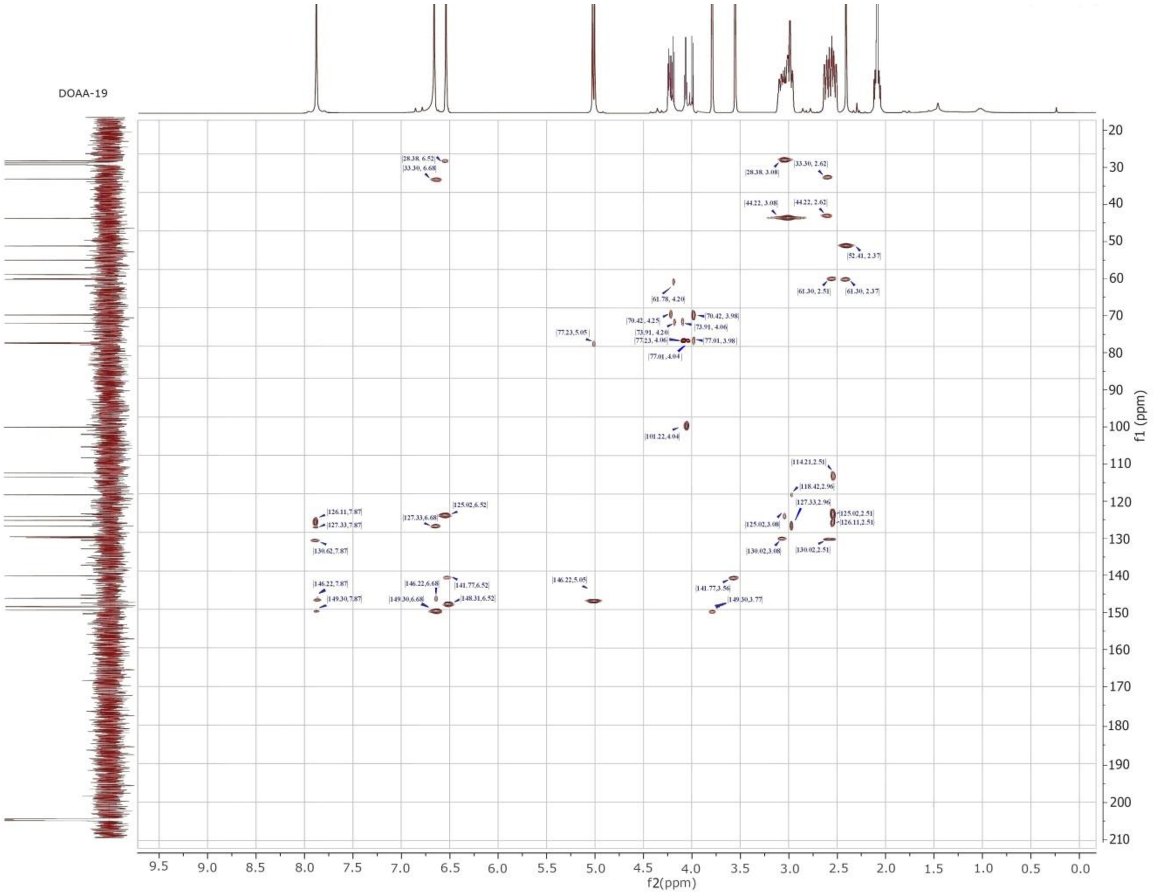

S23: HMBC NMR spectrum of boldine metabolite-**4** (Acetone-*d6*).

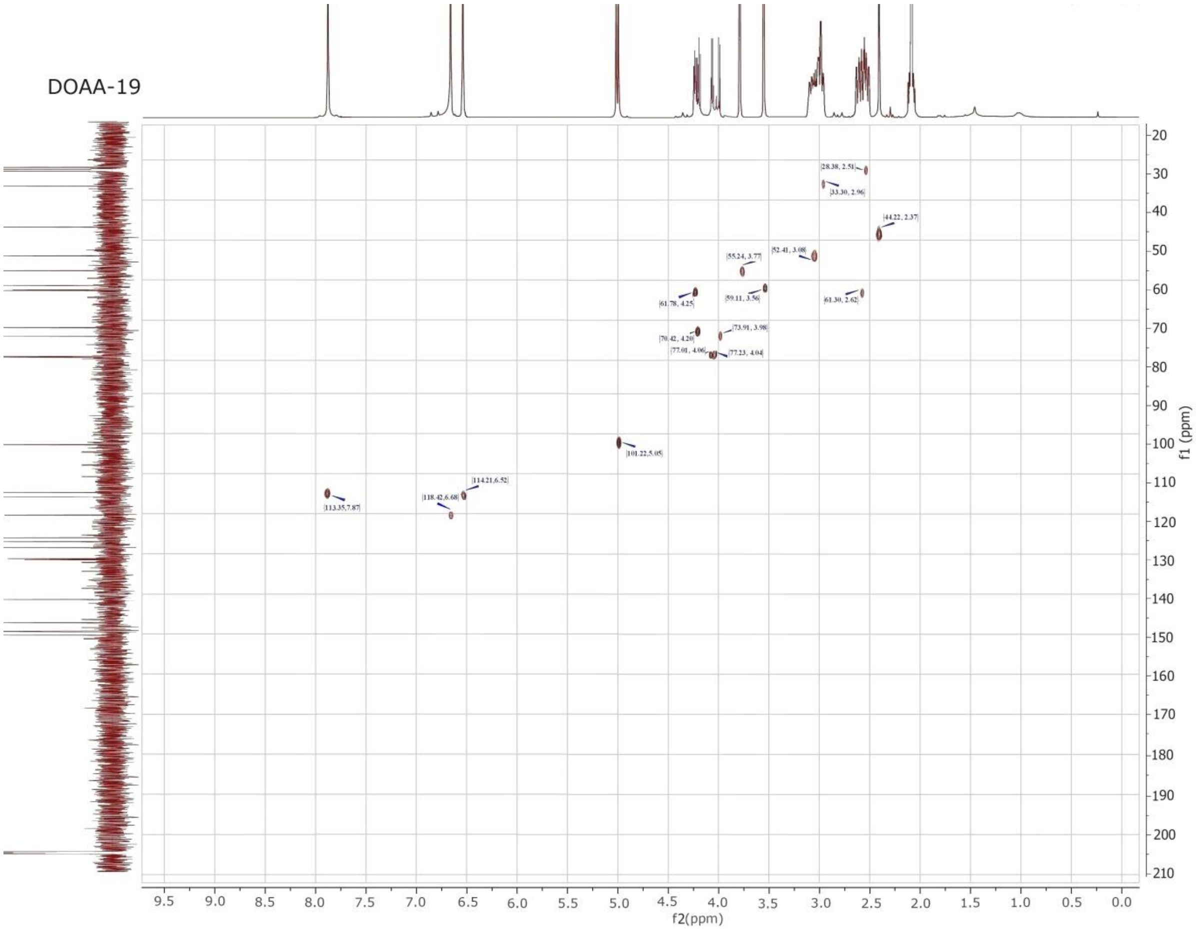

S24: HMQC NMR spectrum of boldine metabolite-**4** (Acetone-*d6*).

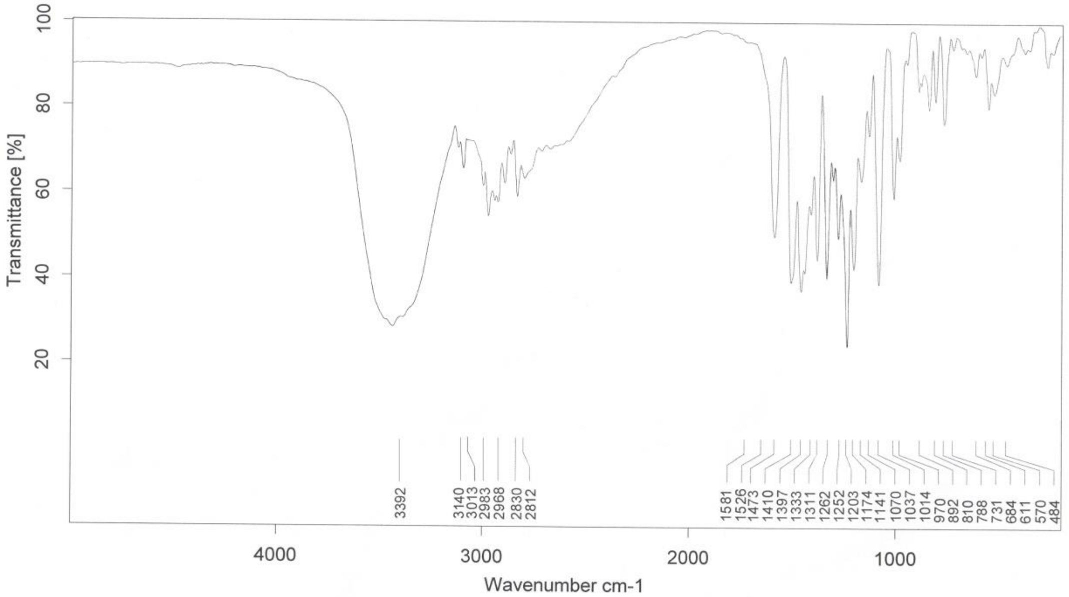

C:\OPUS_7.0.122\MEAS\SAMPLE\Dr.DOAA ELEWA 22-5-2017\19	19	Instrument type and / or accessory

Signature:

S25: IR of boldine metabolite-**4.**

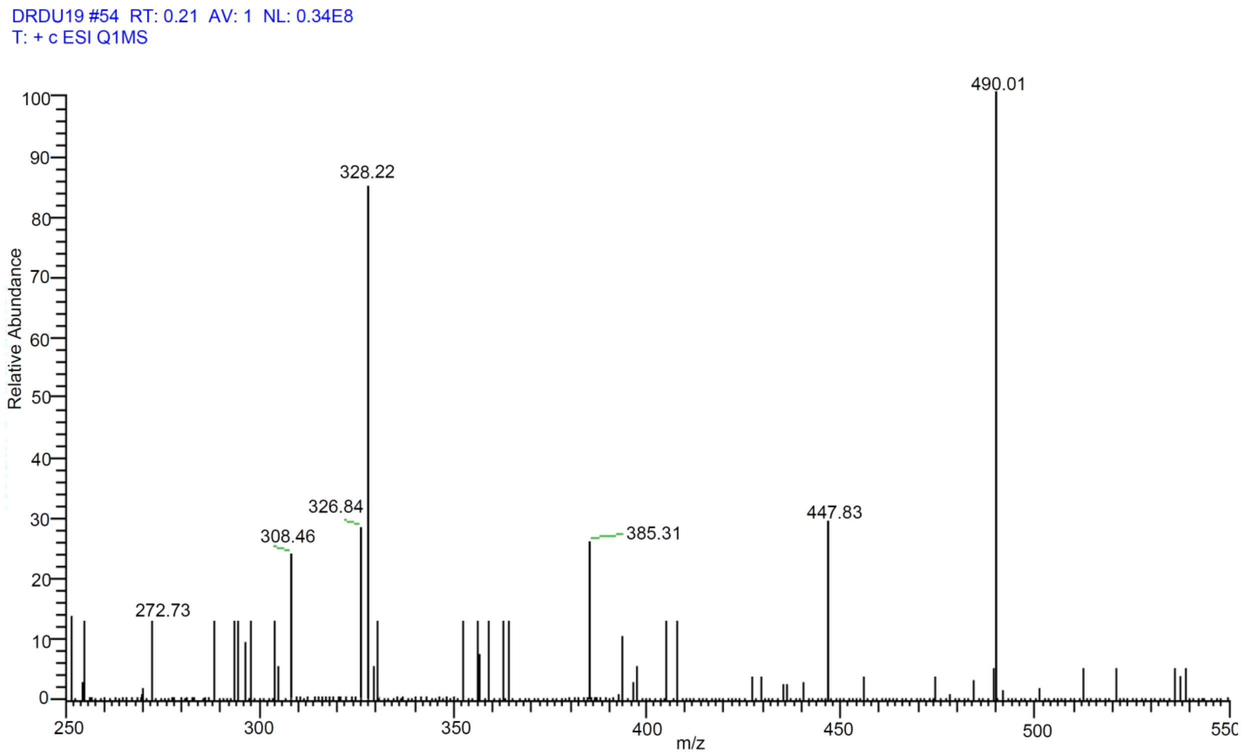

S26: (+) ESI- MS Mass spectrum of boldine metabolite-**4.**

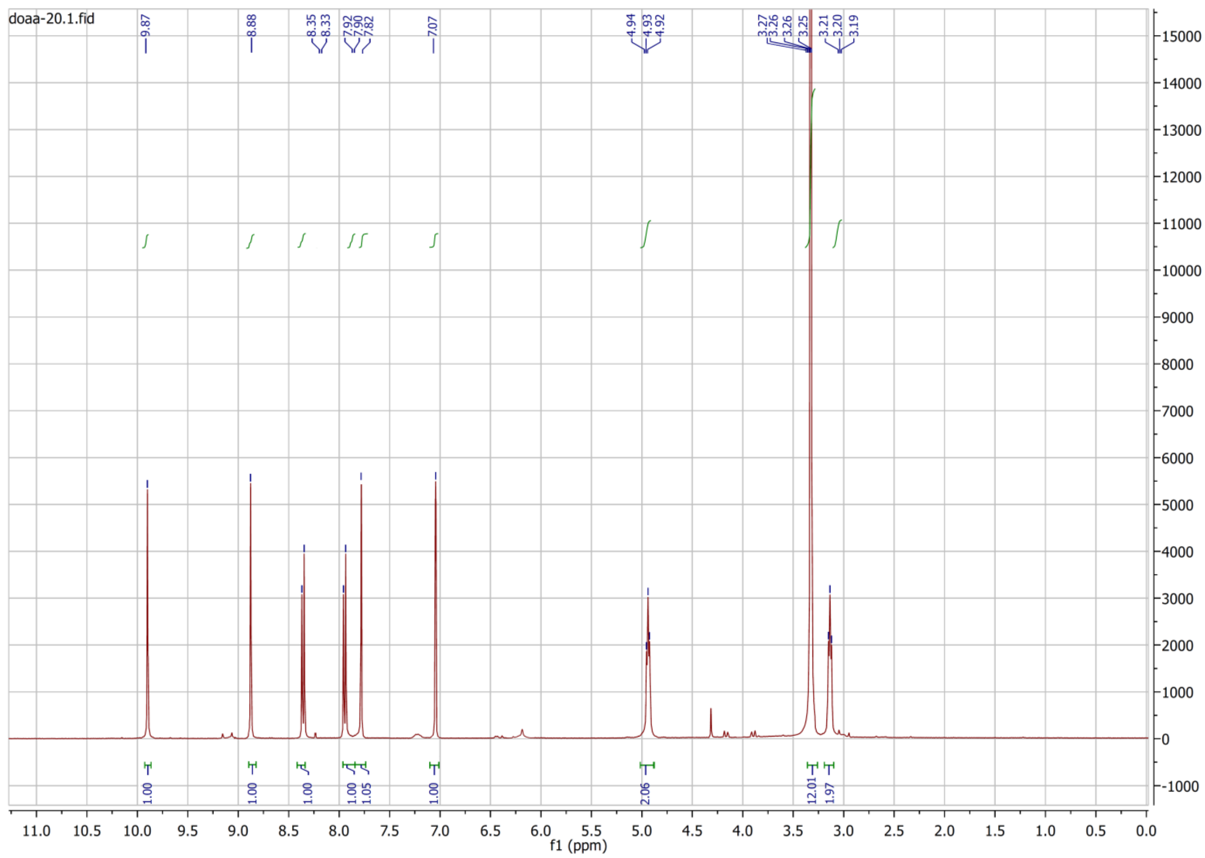

S27: ^{1}H NMR spectrum of berberine metabolite-**5** (400 MHz, MeOD).

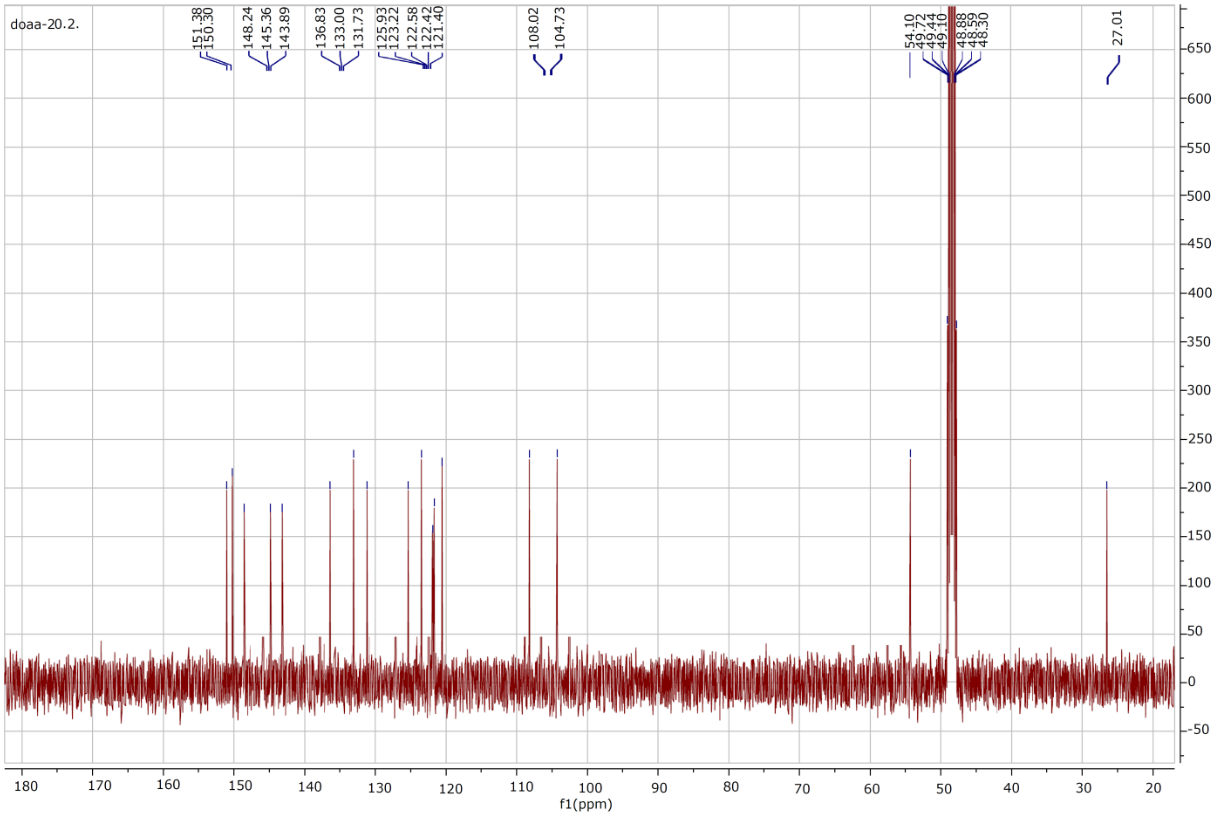

S28: ^{13}C NMR spectrum of berberine metabolite-**5** (100 MHz, MeOD).

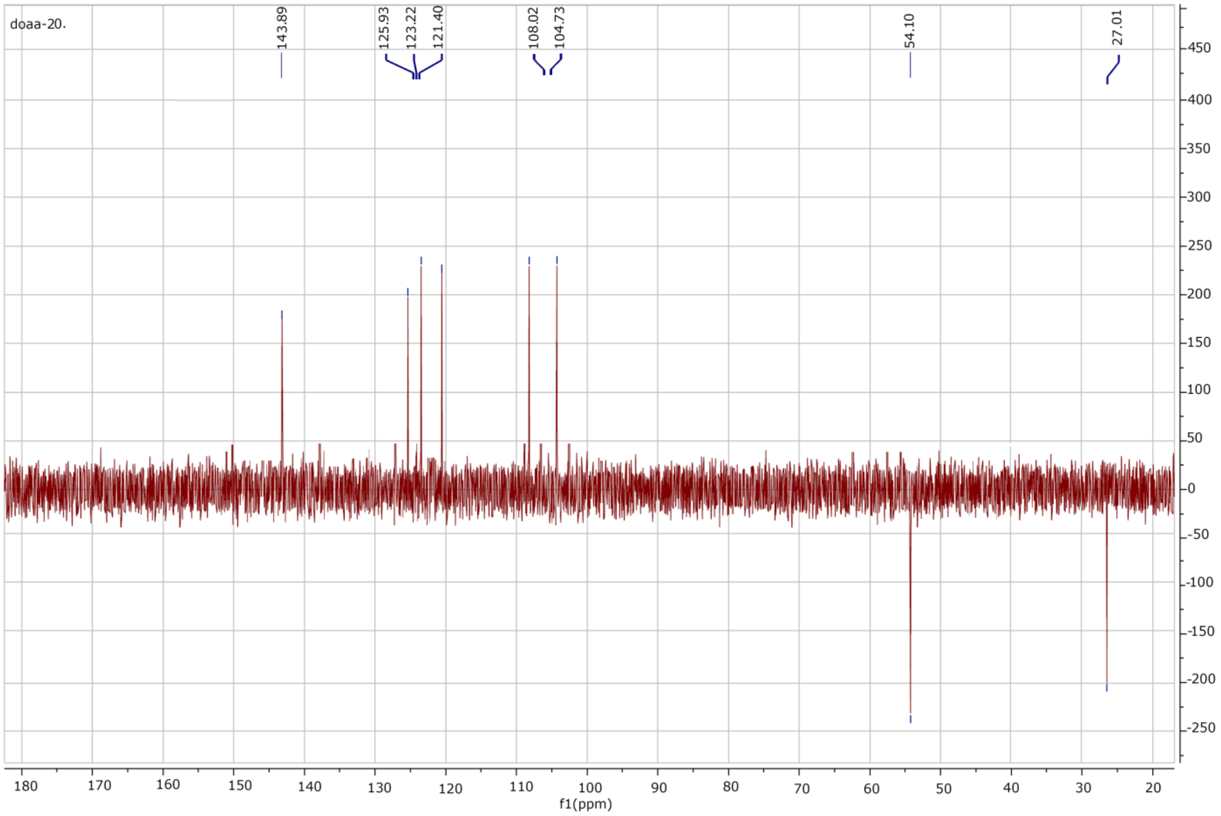

S29: DEPT 135 spectrum of berberine metabolite-**5** (100 MHz, MeOD).

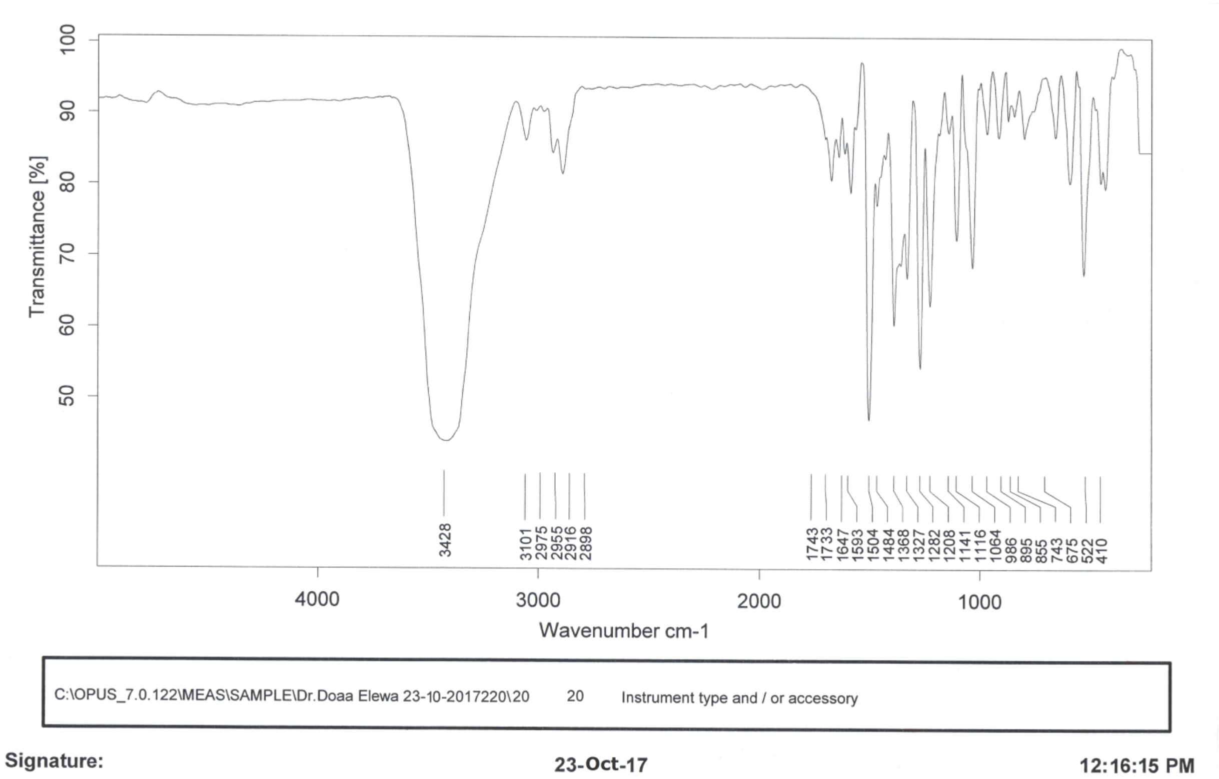

S30: IR spectrum of berberine metabolite-5.

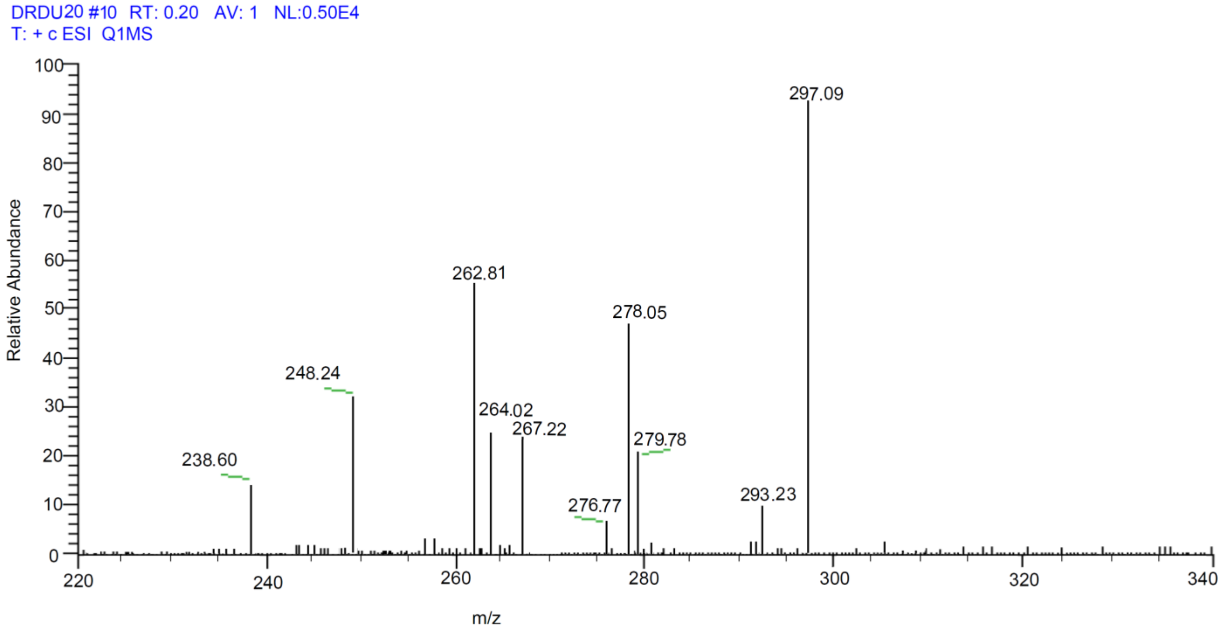

S31: (+) ESI- MS Mass spectrum of berberine metabolite-**5.**

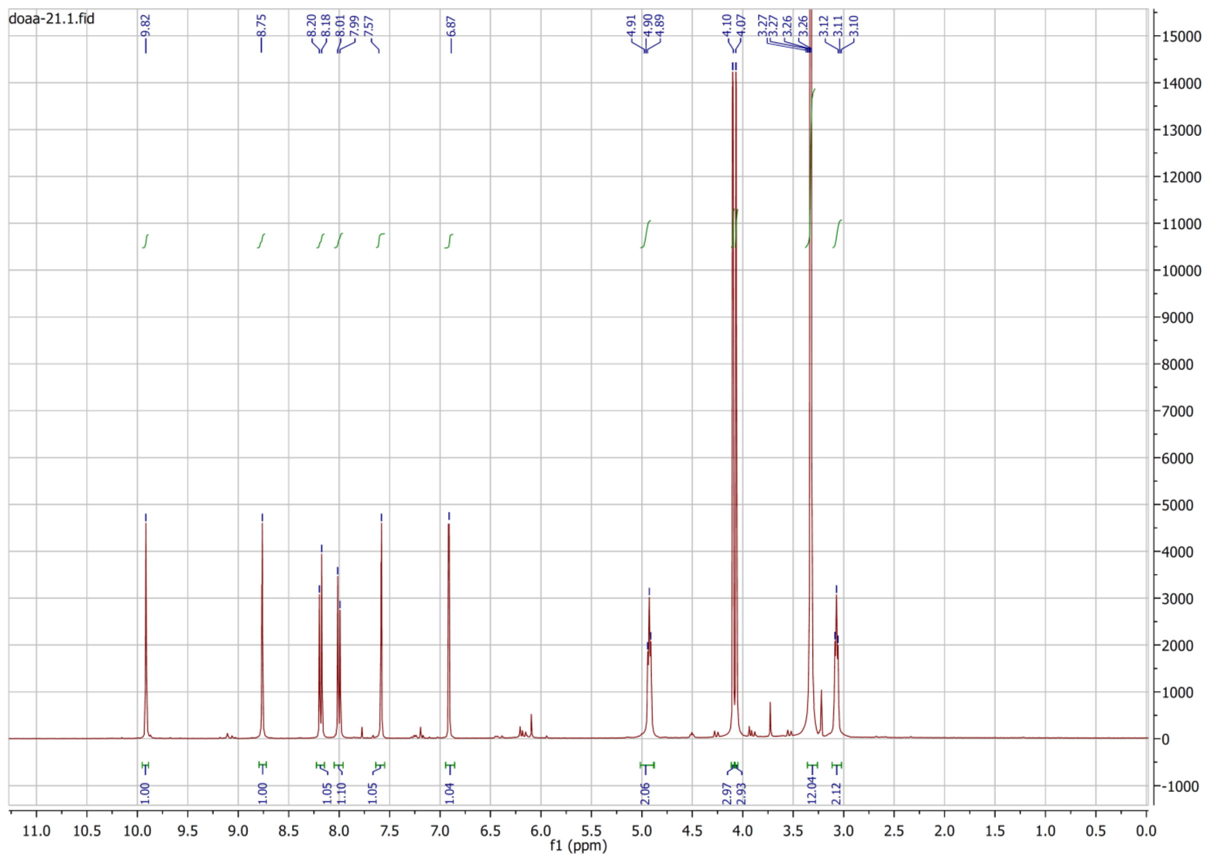

S32: ^{1}H NMR spectrum of berberine metabolite-**6** (400 MHz, MeOD).

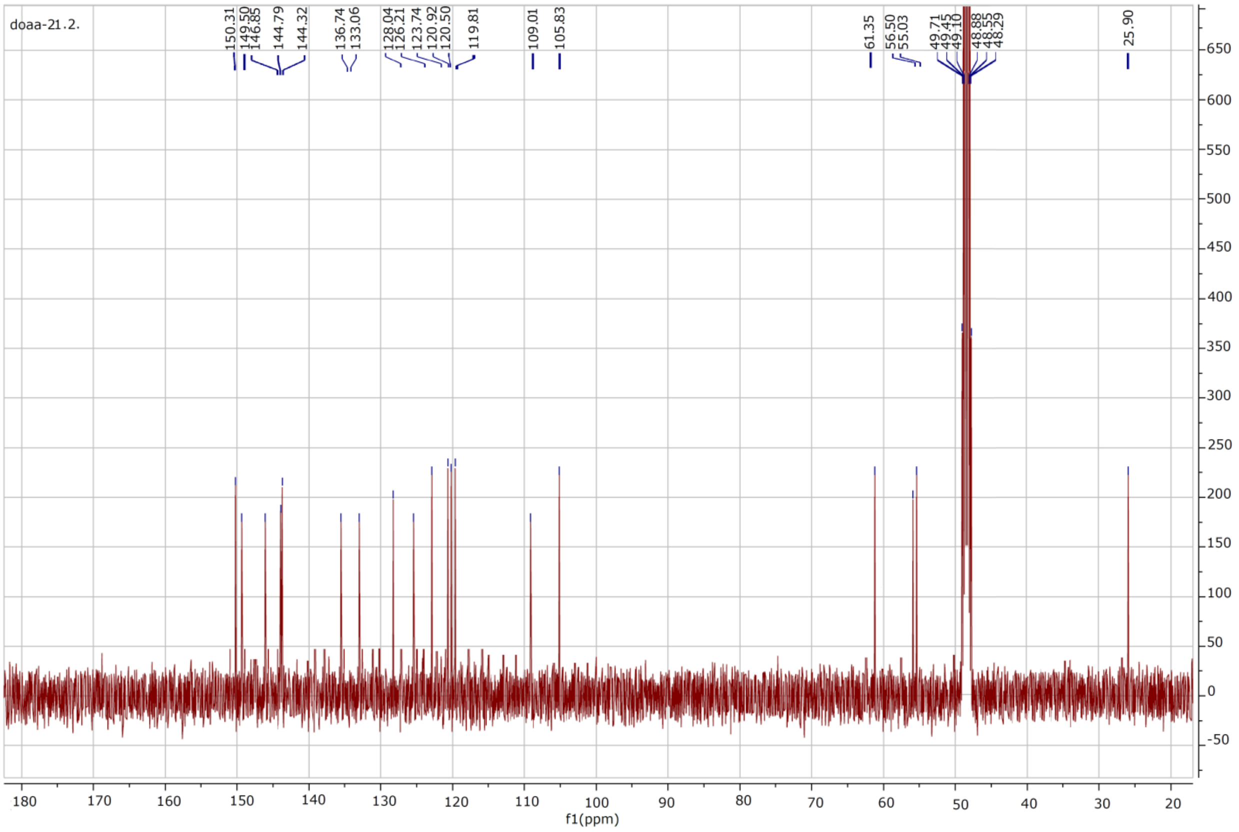

S33: ^{13}C NMR spectrum of berberine metabolite-**6** (100 MHz, MeOD).

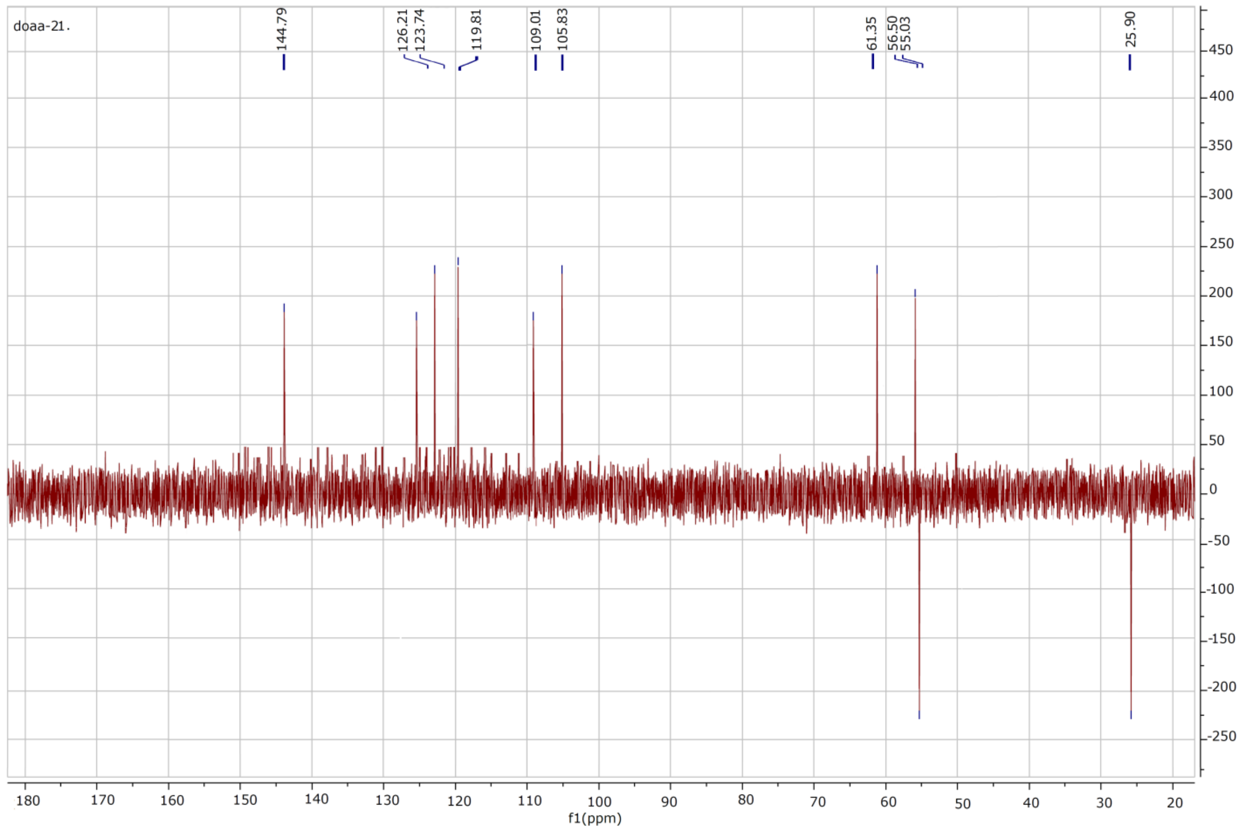

S34: DEPT 135 spectrum of berberine metabolite-**6** (100 MHz, MeOD).

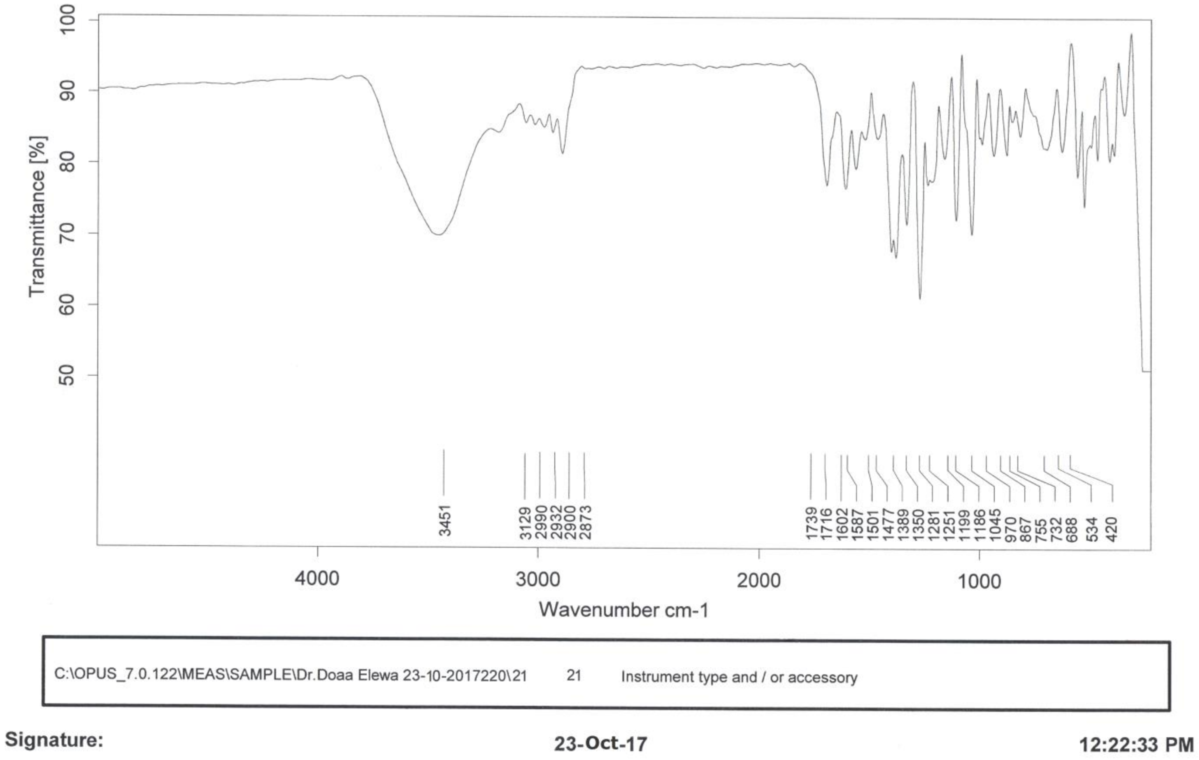

S35: IR spectrum of berberine metabolite-**6**.

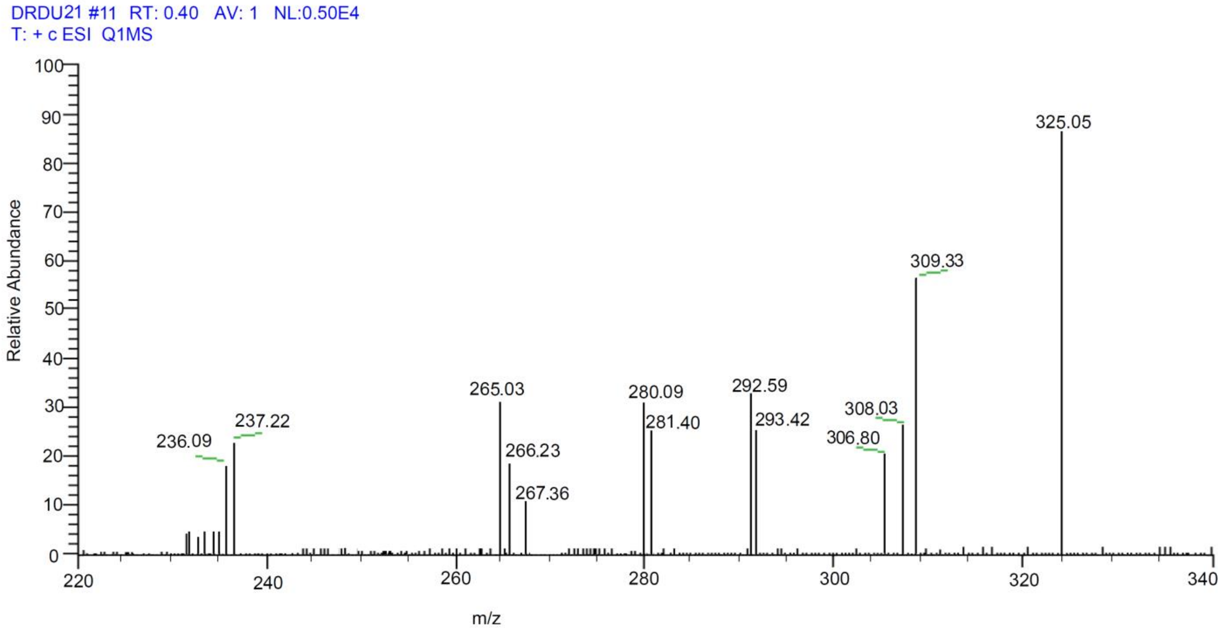

S36: (+) ESI-MS analysis of berberine metabolite-**6.**

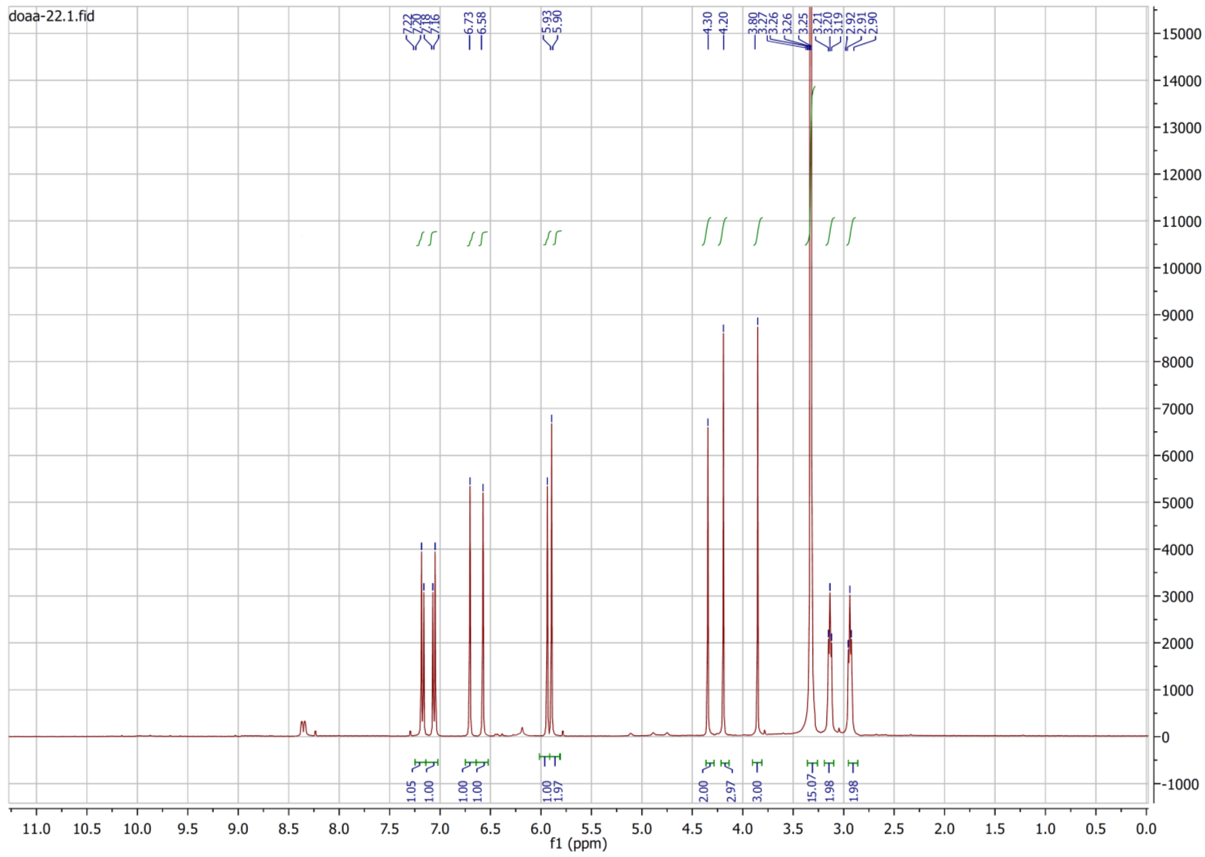

S37: ^{1}H NMR spectrum of berberine metabolite-**7** (400 MHz, MeOD).

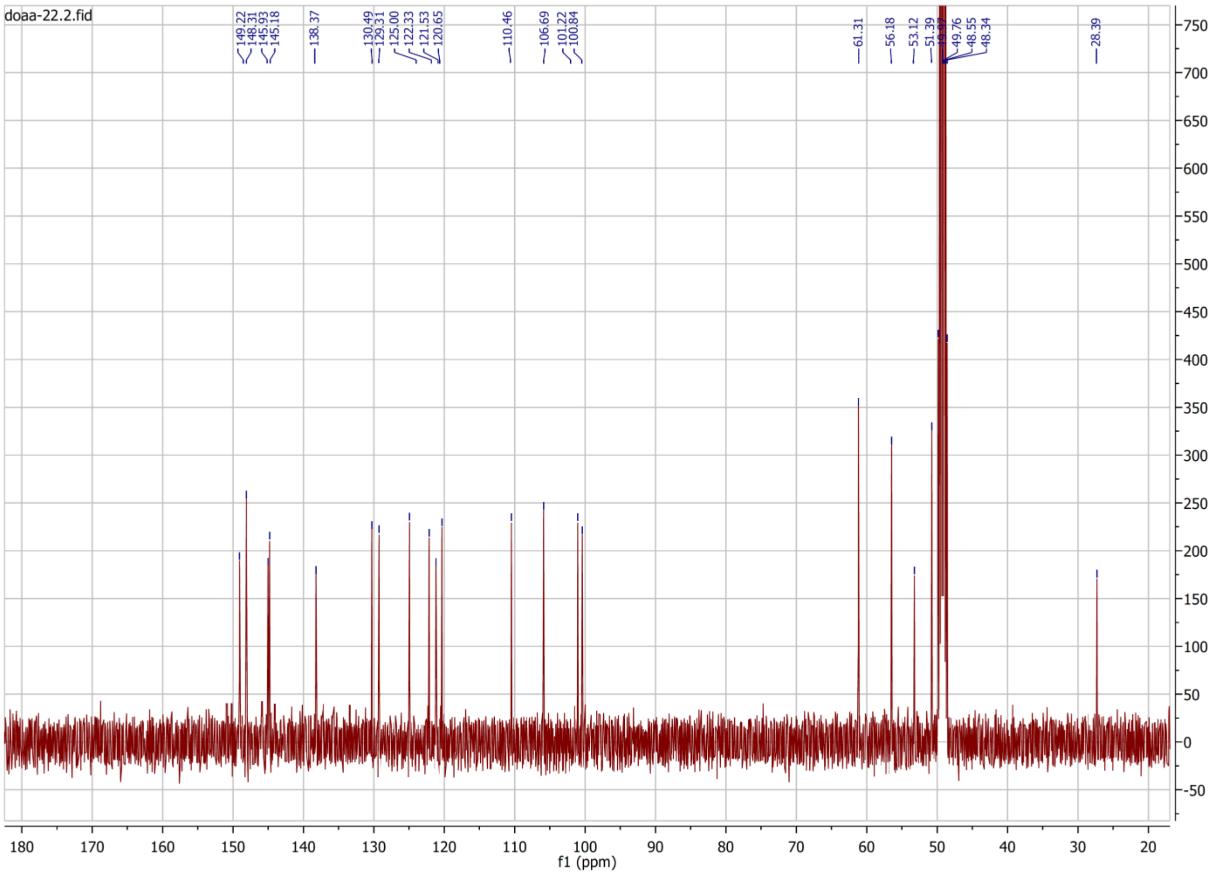

S38: ^{13}C NMR spectrum of berberine metabolite-**7** (100 MHz, MeOD).

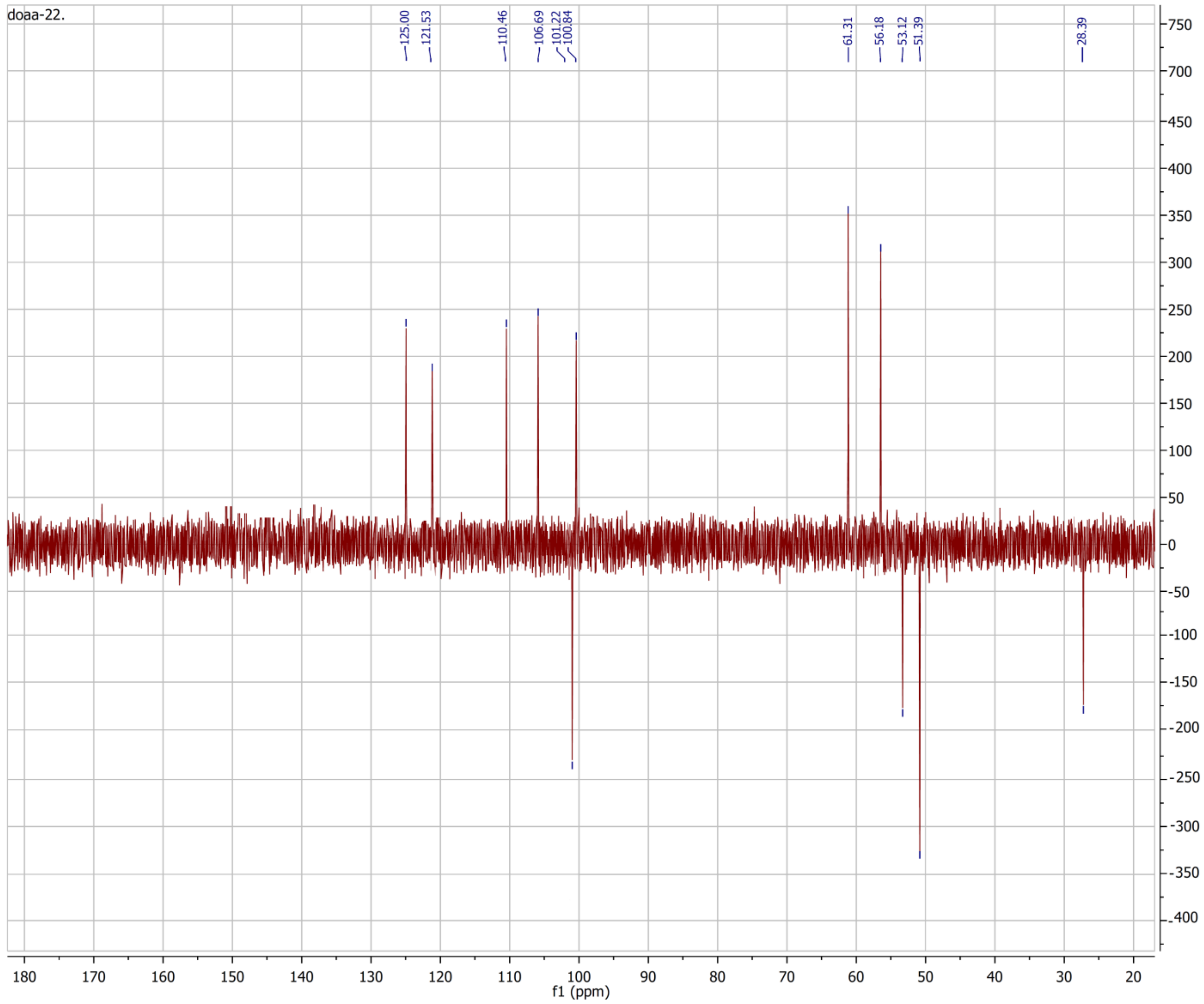

S39: DEPT 135 spectrum of berberine metabolite-**7** (100 MHz, MeOD).

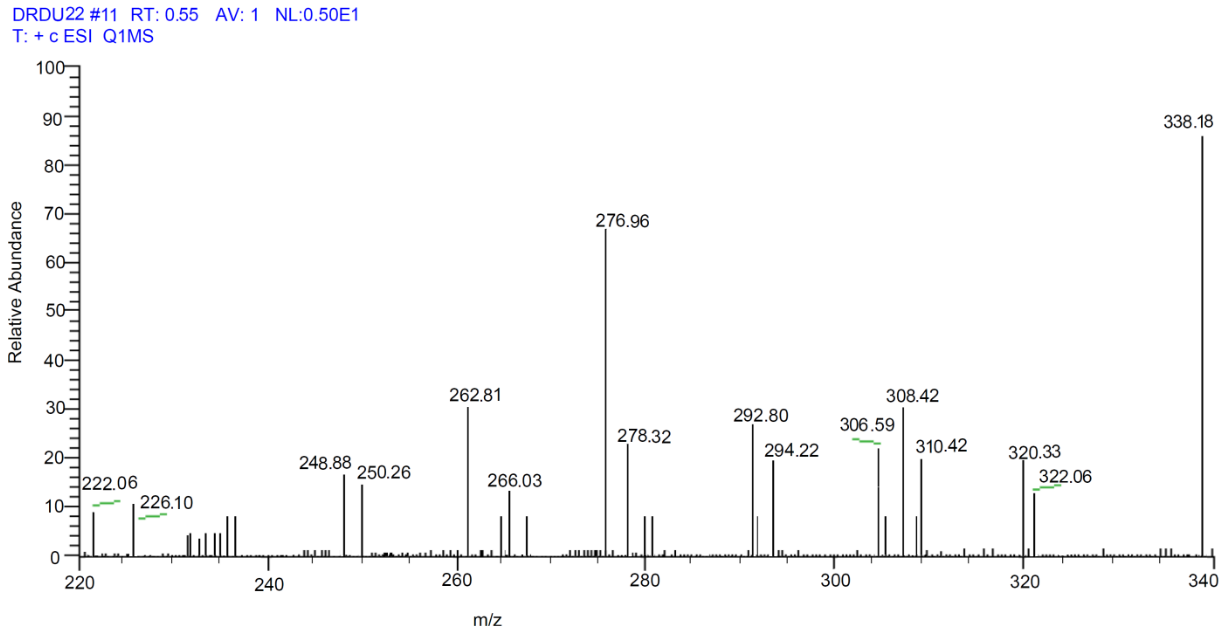

S40: (+) ESI-MS analysis of berberine metabolite-**7.**
